# T cell-inducing vaccine durably prevents mucosal SHIV infection even with lower neutralizing antibody titers

**DOI:** 10.1038/s41591-020-0858-8

**Published:** 2020-05-11

**Authors:** Prabhu S. Arunachalam, Tysheena P. Charles, Vineet Joag, Venkata S. Bollimpelli, Madeleine K. D. Scott, Florian Wimmers, Samantha L. Burton, Celia C. Labranche, Caroline Petitdemange, Sailaja Gangadhara, Tiffany M. Styles, Clare F. Quarnstrom, Korey A. Walter, Thomas J. Ketas, Traci Legere, Pradeep Babu Jagadeesh Reddy, Sudhir Pai Kasturi, Anthony Tsai, Bertrand Z. Yeung, Shakti Gupta, Mark Tomai, John Vasilakos, George M. Shaw, Chil-Yong Kang, John P. Moore, Shankar Subramaniam, Purvesh Khatri, David Montefiori, Pamela A. Kozlowski, Cynthia A. Derdeyn, Eric Hunter, David Masopust, Rama R. Amara, Bali Pulendran

**Affiliations:** 1 Institute for Immunity, Transplantation and Infection, Stanford University School of Medicine, Stanford University, Stanford, CA USA; 2 Department of Pathology and Laboratory Medicine, Emory Vaccine Center, Yerkes National Primate Research Center, Atlanta, GA USA; 3 Department of Microbiology and Immunology, Center for Immunology, University of Minnesota, Minneapolis, MN USA; 4 Department of Microbiology and Immunology, Emory Vaccine Center, Yerkes National Primate Research Center at Emory University, Atlanta, GA USA; 5 Center for Biomedical Informatics, Department of Medicine, Stanford University, Stanford, CA USA; 6 Department of Surgery, Duke University School of Medicine, Durham, NC USA; 7 Department of Microbiology, Immunology, and Parasitology, Louisiana State University Health Sciences Center, New Orleans, LA USA; 8 Department of Microbiology and Immunology, Weill Medical College of Cornell University, New York, NY USA; 9 BioLegend, San Diego, CA USA; 10 Department of Bioengineering, University of California, San Diego, La Jolla, CA USA; 11 3M Corporate Research and Materials Lab, Saint Paul, MN USA; 12 3M Drug Delivery Systems, Saint Paul, MN USA; 13 Department of Medicine, University of Pennsylvania, Philadelphia, PA USA; 14 Department of Microbiology and Immunology, Schulich School of Medicine & Dentistry, The University of Western Ontario, London, Ontario Canada; 15 Department of Pathology, Stanford University School of Medicine, Stanford University, Stanford, CA USA; 16 Department of Microbiology and Immunology, Stanford University School of Medicine, Stanford University, Stanford, CA USA; 17 Present Address: HIV Inflammation and Persistence Unit, Institut Pasteur, Paris, France; 18 Present Address: Pfizer, Andover, MA USA

**Keywords:** Immunology, Diseases, Immunology, Diseases, Immunology

## Abstract

Recent efforts toward an HIV vaccine focus on inducing broadly neutralizing antibodies, but eliciting both neutralizing antibodies (nAbs) and cellular responses may be superior. Here, we immunized macaques with an HIV envelope trimer, either alone to induce nAbs, or together with a heterologous viral vector regimen to elicit nAbs and cellular immunity, including CD8^+^ tissue-resident memory T cells. After ten vaginal challenges with autologous virus, protection was observed in both vaccine groups at 53.3% and 66.7%, respectively. A nAb titer >300 was generally associated with protection but in the heterologous viral vector + nAb group, titers <300 were sufficient. In this group, protection was durable as the animals resisted six more challenges 5 months later. Antigen stimulation of T cells in ex vivo vaginal tissue cultures triggered antiviral responses in myeloid and CD4^+^ T cells. We propose that cellular immune responses reduce the threshold of nAbs required to confer superior and durable protection.

## Abstract (web summary)

An HIV vaccine that elicits both antibodies and cellular immune responses confers long-lasting protection against viral challenge in nonhuman primates.

## Main

The development of a vaccine against HIV infection remains a major global health priority. After more than two decades of intense effort, the RV144 HIV vaccine trial in Thailand raised expectations when it revealed a modest 31% protection against HIV^1^. The RV144 trial was based on a vaccination regimen that involved priming the immune system with a canarypox viral vector (ALVAC-HIV) expressing three HIV proteins (Gag, Pol and the envelope protein Env) followed by booster immunization with recombinant gp120 Env proteins. However, unfortunately the modest success of RV144 could not be reproduced in the HVTN702 trial in South Africa, based on the prime-boost regimen utilized in RV144. This is a major setback for the field, and underscores the need for alternative vaccination strategies in the quest to develop an HIV vaccine. Of note, neither the RV144 nor the HVTN702 vaccine strategy induced nAbs, which have been shown to be effective in preventing simian–human immunodeficiency virus (SHIV) infection in macaques when passively administered^2–4^ and in maintaining viral suppression in chronically infected humans^5,6^.Therefore, learning how to induce robust and persistent nAb responses through vaccination remains a central goal in HIV vaccine research. The recent development of novel immunogens that retain the native trimeric conformation of HIV Env^7,8^ has allowed the generation of autologous nAbs in animal models^9^. In recent studies, immunization of rhesus macaques with BG505 SOSIP.664, the prototype native-like Env trimer, elicited autologous nAbs^10^ that, if present above a threshold titer of 500, were protective against repeated intrarectal challenge with autologous SHIV^11^. However, inducing such high titers of nAbs that persist for long periods is a demanding task and has not been achieved with HIV Env immunogens. Therefore, vaccination approaches that can lower the threshold of protective nAb titers are highly desirable.

CD8^+^ T cells represent the complementary arm of the immune system that effectively controls viral replication during natural infection^12^ and in vaccination settings^13^. Historically, successful vaccines such as those against smallpox and yellow fever induce strong and long-lasting CD8^+^ T cell responses^14,15^ providing a rationale to include these responses in an HIV vaccine design. The concept of a CD8^+^ T cell vaccine was tested with the use of a recombinant adenovirus serotype 5 (Ad5) vector, expressing HIV antigens in the phase 3 STEP trial in which no protection was seen^16^. Nonetheless, interest in CD8^+^ T cell-based vaccines has been renewed by recent studies in macaques, demonstrating unprecedented clearance of SIVmac239 by atypical, broad CD8^+^ T cell responses induced by a cytomegalovirus-based vaccine^13,17,18^. Moreover, strong CD8^+^ T cell responses at the port of viral entry, such as vaginal mucosa, could offer enhanced protection in the presence of nAbs^19^. Such mucosal-homing CD8^+^ tissue-resident memory T cells (TRMs) can be elicited by sequential immunization with heterologous vectors expressing the same antigen^20,21^. We recently showed that vaccination of macaques sequentially with three heterologous viral vectors (HVVs) expressing SIVmac239 Gag (vesicular stomatitis virus (VSV)-Gag, vaccinia virus (VV)-Gag and Ad5-Gag) induced a high magnitude of Gag-specific CD8^+^ effector T cell and TRM responses^22^. In this study, although CD8^+^ T cell responses alone were insufficient for protection against mucosal SHIV challenge, there was limited protection in younger animals when CD8^+^ T cell responses were combined with Env-specific binding antibody responses^22^.

In the current study, we determined whether immunization with HVVs expressing SIVmac239 Gag in concert with native-like BG505 SOSIP.664 trimer, which induces nAbs, could confer enhanced protection against intravaginal infection with the homologous SHIV-BG505 virus. We immunized BG505 SOSIP.664 adjuvanted with the TLR7/8 ligand 3M-052 encapsulated in polylactic glycolic acid (PLGA) nanoparticles (SOSIP/3M-052), which induced high autologous nAb titers, and provided protection from ten low-dose vaginal challenges. Of note, in animals immunized with SOSIP/3M-052, a serum autologous nAb titer >300 was associated with protection. In contrast, in the cohort immunized with HVVs in concert with SOSIP/3M-052, many animals with nAb titers <300 were protected. In these animals, Env-specific vaginal binding antibody titers correlated with protection. Antigen stimulation of T cells in ex vivo vaginal tissue cultures triggered antiviral responses in myeloid and CD4^+^ T cells, suggesting that T cells in vaginal mucosa can stimulate local antiviral immunity, which may synergize with antibodies to offer enhanced resistance to HIV.

## Results

### Immunization with SOSIP/3M-052 induces robust autologous neutralizing antibody responses

**Fig. 1:**
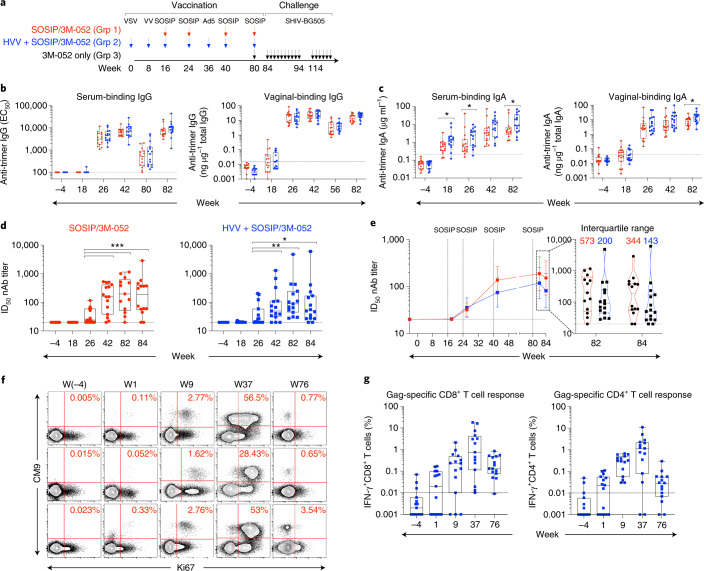
Immunization with SOSIP/3M-052 induces robust antibody responses. **a**, Schematic representation of immunization groups (Grp) and regimens. The arrows (color coded as indicated) denote when various immunizations were given, with the time in weeks also recorded. **b**,**c**, Anti-trimer IgG- and IgA-binding antibodies measured in serum and vaginal secretions of vaccinated animals collected at the indicated time points. The asterisks represent significant differences between the groups at a time point as measured by Mann–Whitney rank-sum test (two-tailed, *P < 0.05). EC_50_, half-maximum effective concentration. **d**, Serum nAb ID_50_ titers to BG505.T332N pseudovirus. The asterisks represent statistically significant differences between time points as measured by a Wilcoxon matched-pairs signed-rank test (two-tailed, *P < 0.05, **P < 0.01 and ***P < 0.001). **e**, A line plot to compare the magnitude of autologous nAb ID_50_ titers between SOSIP/3M-052 and HVV + SOSIP/3M-052 groups. The violin plots on the right show the distribution of responses at 2 and 4 weeks after the last SOSIP/3M-052 immunization (at weeks 82 and 84). Each symbol represents an individual animal and the median values are indicated by black dotted horizontal lines. The 25th and 75th percentiles are indicated by the colored (red or blue) dashed lines. **f**, Flow cytometry plots representing Gag-CM9 tetramer and Ki67 expression by CD8^+^ T cells (gated as live CD3^+^CD8^+^ singlets) in blood collected at time points indicated on the top. Data from the three Mamu-A*01 animals in the HVV + SOSIP/3M-052 group are shown. **g**, Gag-specific CD8^+^ and CD4^+^ T cell responses measured in blood at the time points (baseline, 1 week after each vaccination and 8 weeks before challenge) indicated on the *x* axis are plotted. The percentage Gag-specific IFN-γ response was calculated by subtracting the frequency of IFN-γ^+^ T cells in the DMSO-treated cells from the values recorded after stimulation with the Gag peptide pool. In **b**–**g**, sample sizes were *n* = 15 per group at all time points, except week 82; at week 82, *n* = 13 in the SOSIP/3M-052 group because serum could not be collected from two animals. In box plots, each symbol represents an animal. The box shows median, upper and lower quartiles. The whiskers show 5th to 95th percentiles. Red and blue colors indicate immunization groups 1 and 2, respectively.

Forty-five female rhesus macaques (Supplementary Table 1) were distributed into three groups of 15 animals each. Animals in group 1 received four consecutive immunizations of BG505 SOSIP.664 adjuvanted with the TLR7/8 ligand 3M-052 encapsulated in PLGA nanoparticles (SOSIP/3M-052)^22^. Animals in group 2 were vaccinated via a sequential regimen involving HVVs comprising VSV, VV and Ad5-expressing SIVmac239 Gag in concert with SOSIP/3M-052 administered at the corresponding time points used in group 1 (Fig. 1a). The SOSIP/3M-052 immunizations were administered subcutaneously at weeks 16, 24, 40 and 80 in two sites, whereas the VSV, VV and Ad5 viral vectors were administered intravenously at weeks 0, 8 and 36, respectively. Control animals in group 3 were administered one dose of 3M-052 in PLGA nanoparticles 4 weeks before the first viral challenge (Fig. 1a).

In groups 1 and 2, serum anti-trimer IgG-binding antibodies were only quantifiable after the second SOSIP/3M-052 immunization; the titers increased marginally after the third immunization and were no higher after the fourth (Fig. 1b). Notably, the anti-trimer IgG titers decreased less than tenfold in the 40-week period between the third and fourth immunizations, which is consistent with previous studies^22,23^. Like the serum response, the vaginal IgG response was also detected at high levels only after the second immunization and peaked after the third immunization (Fig. 1b). Serum and vaginal IgA anti-trimer antibodies were also detected (Fig. 1c). While the IgG responses were comparable between the immunization groups in both compartments, the IgA responses were moderately higher in the HVV + SOSIP/3M-052 group at some time points.

**Extended Data Fig. 1:**
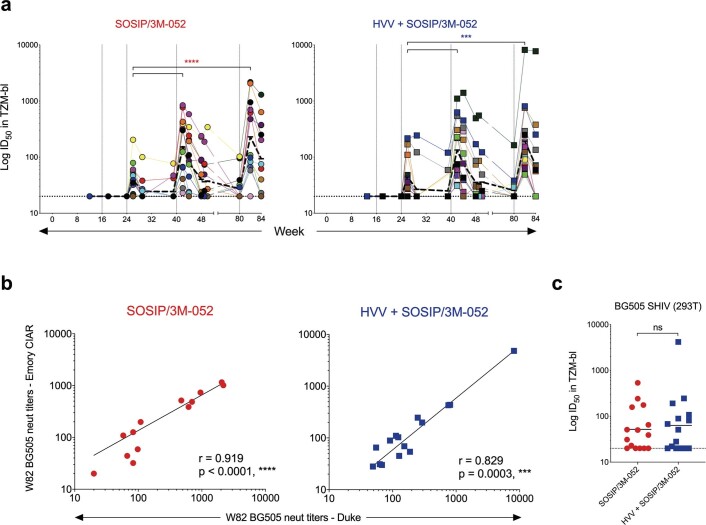
Assessment of serum autologous nAb titers. **a**, Kinetics of serum nAb titers measured against BG505.T332N pseudovirus measured in the Duke Central Laboratory. Each symbol represents an individual animal. The dark discontinuous lines show geometric mean titers. The asterisks represent statistically significant differences between time points as measured by the Wilcoxon matched-pairs signed rank test (two-tailed, ***p = 0.0006 and ****p < 0.0001). **b**, Spearman’s correlation between autologous nAb titers measured in week 82 sera by the Duke and Emory laboratories. In correlation plots, r and p represent Spearman’s r and two-tailed p values. **c**, NAb ID50 titers in week 84 sera measured against replication-competent SHIV-BG505 (Emory laboratory) produced in HEK293T cells. Geometric means are indicated. Statistical differences were analyzed using two-tailed Mann-Whitney rank sum test (ns, p = 0.96). n = 15 per group in all the panels except week 82 at which n = 13 in the SOSIP/3M-052 group).

SOSIP/3M-052 vaccination induced robust half-maximum infectious dose (ID_50_) titers of autologous nAbs to BG505.T332N Env pseudovirus (Fig. 1d,e). We first analyzed sera from baseline and peak time points, 2 weeks after each vaccination. In both vaccine groups, nAb titers were first detected after the second immunization (week 26) and were significantly boosted after the third (Fig. 1d). The geometric mean titers increased from 1:32 at week 26 to 1:137 at week 42 (2 weeks after second and third protein vaccinations, respectively) in group 1, whereas for group 2 the corresponding values were 1:37 and 1:74 (Fig. 1d). The third booster vaccination 40 weeks later increased geometric mean titers to 1:188 (range 20–1,157) and 1:118 (28–4,810) in groups 1 and 2, respectively. Although there was no statistically significant difference in the magnitude of autologous nAb titer between vaccination groups at any time point, the distribution of titers among individual animals was noticeably different. The interquartile range, a measure of scatter, was ~2.5-fold higher in group 1 than in group 2 (Fig. 1e). In other words, the magnitude of nAb titer for the upper 50-percentile animals in group 1 was 2.5-fold higher than in corresponding group 2 animals. Sera from the same and some additional time points were also assessed in the Duke Central Laboratory to document the durability of nAb response and to be able to compare titers with other ongoing studies involving 3M-052-adjuvanted vaccination with SOSIP (S.P.K.*,* manuscript in preparation). As expected, the two datasets were highly consistent and correlated (Extended Data Fig. 1a,b). The nAb titers declined substantially in both groups within 8 weeks after the third SOSIP/3M-052 immunization, and only a few animals had detectable titers on the day of the fourth immunization 40 weeks later (at week 80) (Extended Data Fig. 1a). We also measured nAb titers against the replication-competent SHIV-BG505 virus produced in HEK293T cells, using a molecular clone, in week 84 serum. The titers were about threefold lower in comparison to the data derived using BG505.T332N pseudovirus, but the median titers did not differ significantly between the groups (Extended Data Fig. 1c).

**Extended Data Fig. 2:**
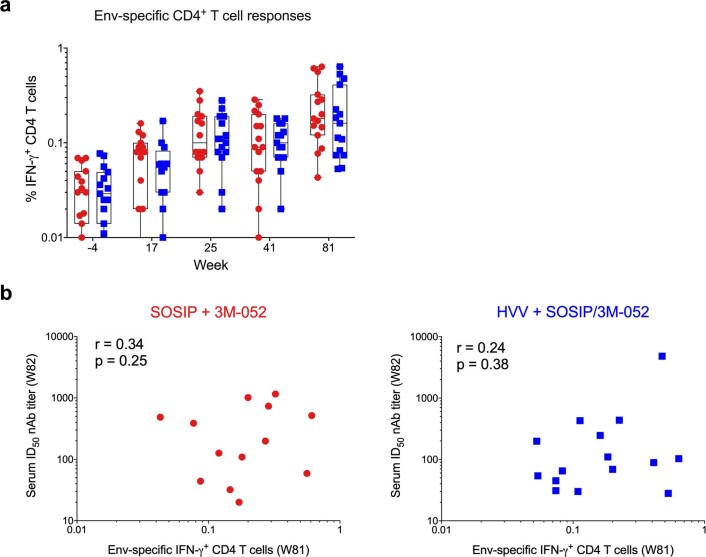
Env-specific CD4 T cell responses. **a**, Env-specific CD4^+^ T cell responses measured in blood at the time points (Baseline and 1 week after each vaccination) indicated on the X-axis are plotted. Each symbol represents an animal. The box shows median, upper and lower quartiles, and the whiskers represent 5–95 percentiles (n = 15 in each group). The %Env-specific IFN-γ response was calculated by subtracting the frequency of IFN-γ^+^ T cells in the DMSO-treated cells from the values recorded after stimulation with the Env peptide pool. **b**, Spearman’s correlation between serum autologous nAb ID_50_ titers and Env-specific IFN-γ^+^ CD4 T cells. The red circles and blue squares represent SOSIP/3M-052 (n = 13) and HVV + SOSIP/3M-052 (n = 15) groups, respectively. The r and p values represent Spearman’s r and two-tailed p values.

**Extended Data Fig. 3:**
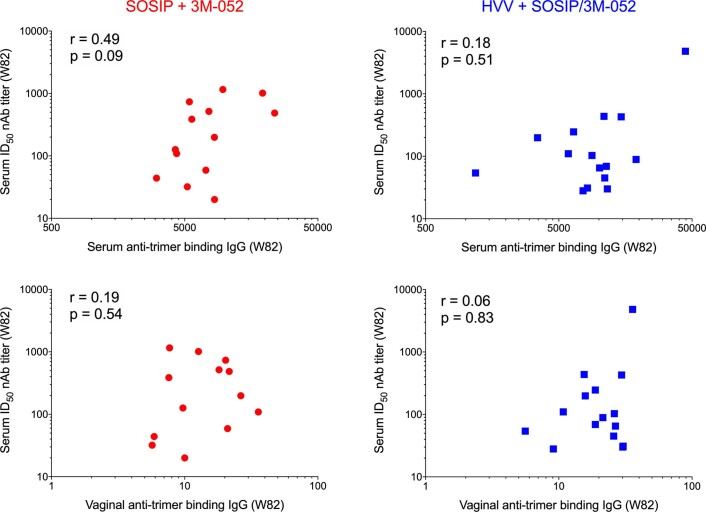
Correlation of serum nAb titers with serum and vaginal trimer-binding titers. Spearman’s correlation between serum autologous nAb ID_50_ titers at week 82, the peak time point of the response, and serum binding IgG titers (upper panels) and vaginal binding IgG titers (lower panels), at the corresponding time points. SOSIP/3M-052 (n = 13) and HVV + SOSIP/3M-052 (n = 15) immunization groups are indicated by red circles and blue squares, respectively. The r and p values represent Spearman’s r and two-tailed p values.

In addition to potent antibody responses, SOSIP/3M-052 immunizations induced modest CD4^+^ T cell responses (Extended Data Fig. 2a). There was no CD8^+^ T cell response, as seen previously^22^. Of note, HVV immunizations did not impact CD4^+^ T cell responses to Env immunization. Furthermore, there was no correlation between Env-specific CD4^+^ T cells and nAb responses (Extended Data Fig. 2b) or between binding and neutralizing antibodies (Extended Data Fig. 3) consistent with recent studies^10^.

### Immunization with HVV induces high magnitude of Gag-specific T cells and TRMs

**Extended Data Fig. 4:**
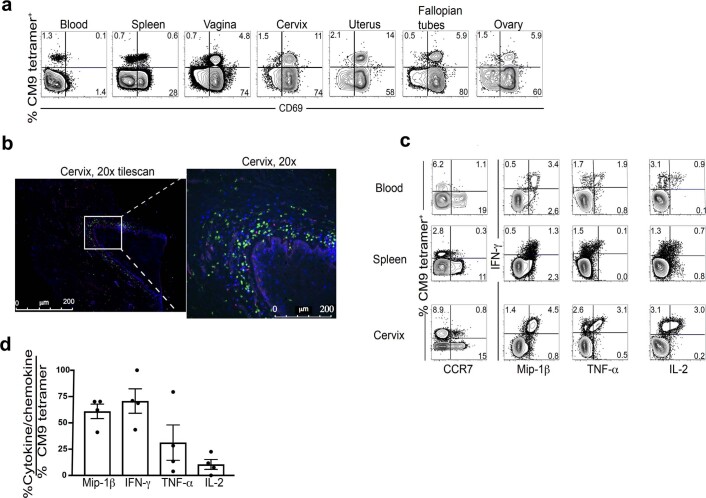
HVV vaccination establishes abundant, functional Gag-specific CD8+ T cells with a resident memory phenotype in the female genital tract. **a**, Four Mamu-A*01^+^ animals receiving HVV vaccinations (from the previous study^22^) were necropsied >100 days post Ad5-Gag vector administration. Lymphocytes isolated from blood, spleen, vagina, cervix, uterus, fallopian tubes and ovaries were analyzed by flow cytometry. CD8^+^ T cells were gated as CD20^−^, CD3^+^, CD4^−^, CD8^+^ cells. **b**, Immunohistochemical staining of fresh cervical tissue as in **a** was incubated with Gag-CM9 tetramer, snap frozen and sectioned. The sections were stained with CD8 and CD4 antibodies. Gag-CM9 tetramer is shown in red, CD8 in blue and CD4 in green. **c**, Representative flow cytometry plots of direct ex vivo Gag-CM9 tetramer staining (left column) and in vitro cytokine stimulation with CM9 peptide (right three columns) of blood, spleen and female genital tract lamina propria CD8^+^ T cells gated as in **a**. Analyses in panels **b** and **c** were a representative of analysis from 4 independent animals. **d**, Ratio of frequency of cytokine^+^ or chemokine^+^ cells (mean ± SEM) after in vitro peptide stimulation to Gag-CM9^+^ cells after direct ex vivo stain from all animals as in **a**.

The immunogenicity of HVV vaccinations was evaluated by measuring Gag-specific CD8^+^ and CD4^+^ T cell responses in blood 1 week after each vaccination, the peak time point as found in our previous study^22^, as well as at a later time point (week 76) to assess durability of response. Gag-CM9 tetramer-positive cells increased after each viral vector immunization and reached remarkably high levels, up to 56% after Ad5-Gag administration in the three Mamu-A*01 animals (limited to Mamu-A*01 animals due to availability of tetramers) in the cohort (Fig. 1f). Furthermore, T cell responses, especially CD8^+^ T cells, were highly durable and persisted up to week 76 (10 months after Ad5-Gag vaccination) with detectable interferon (IFN)-γ^+^CD8^+^ T cells (Fig. 1g). HVV vaccination also induced a strong Gag-specific IFN-γ^+^CD4^+^ T cell response in blood (Fig. 1g). In addition, our previous work has demonstrated that vaccination with HVV can induce robust TRM responses in the vagina and cervix^22^. In the current study, we did not collect vaginal biopsies before challenge, as the primary goal of this study was to assess protection and we wished to avoid local trauma and inflammation at the site of infection. However, we were able to confirm the presence of CD8^+^ TRMs in multiple tissues using samples from the previous study^22^. There were abundant Gag-specific CD8^+^ T cells in blood, spleen and multiple parts of the female genital tract >100 d after Ad5-Gag immunization (Extended Data Fig. 4a,b). We tested the potential function of TRMs by stimulating them with CM9 peptide and observed that vaccine-elicited Gag-CM9-specific memory CD8^+^ T cells produced abundant levels of IFN-γ, macrophage inflammatory protein (MIP)-1β, tumor necrosis factor-α and interleukin (IL)-2 and expressed low levels of CCR7 (Extended Data Fig. 4c,d).

### Both vaccines induce significant protection against repeated vaginal infection with SHIV

**Fig. 2:**
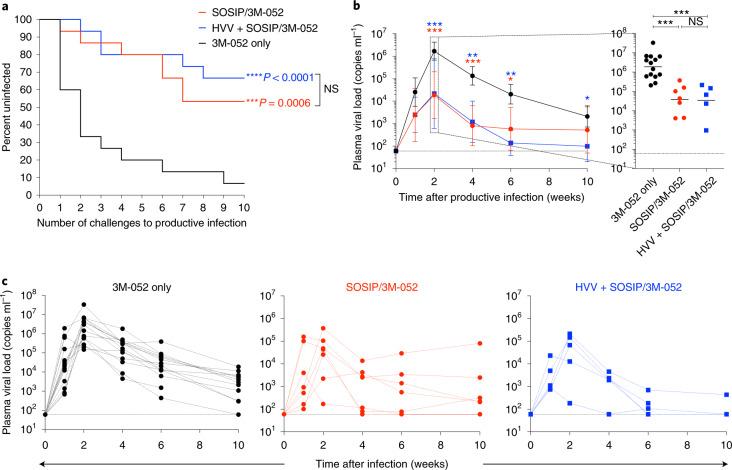
Protection against intravaginal low-dose autologous SHIV challenge. **a**, Kaplan–Meier survival curves showing the fraction of uninfected animals following each challenge (*n* = 15 in each group). The ‘number of challenges to productive infection’ used in the *x* axis was defined as the time point 2 weeks before the first detection of viremia. *P* values (two-tailed) denote statistically significant differences calculated by log-rank (Mantel–Cox) test (NS, not significant, *P* = 0.56). **b**, Plasma viral load at various time points after infection (geometric mean ± s.e.m.), *n* = 14, 7 and 5 in 3M-052 only, SOSIP/3M-052 and HVV + SOSIP/3M-052 groups, respectively. The week before the first detection of viremia was considered as W0. The peak viral load for each of the infected animals is shown on the right. Asterisks denote statistically significant differences in comparison to the 3M-052 control (black) group, as measured by Mann–Whitney rank-sum test (two-tailed, ***P < 0.001, **P < 0.01 and *P < 0.05). **c**, Longitudinal viral load profile for each infected animal is shown.

The primary end point of the study was protection against ten repeated low-dose vaginal challenges with the autologous SHIV-BG505, a tier 2 virus with neutralization resistance properties representative of circulating HIV-1 strains, produced in rhesus peripheral blood mononuclear cells (PBMCs)^24^. To this end, 1 month after the final SOSIP/3M-052 immunization, we challenged the animals weekly, in total ten times. If the plasma viral load was positive (>60 plasma viral RNA copies ml^−1^) for 2 consecutive weeks, then the animal was defined as infected and not further challenged. In comparison to the control arm that received only 3M-052, both vaccine regimens offered significant protection (Fig. 2a). While 80% of control animals were infected within four challenges, 8 of the 15 animals in the SOSIP/3M-052 group (53.3% protection, *P* = 0.0006) and 10 of the 15 animals in the HVV + SOSIP/3M-052 group (66.7% protection, *P* < 0.0001) remained protected after ten challenges. Furthermore, in those animals that were infected, the peak viremia in blood was lower by more than tenfold in vaccinated animals (Fig. 2b). Notably, the plasma viral load at the peak in the SOSIP/3M-052 group (1 × 10^5^ mean copies ml^−1^) was as low as in the HVV + SOSIP/3M-052 group (0.9 × 10^5^ mean copies ml^−1^), reflecting the ability of nAbs to curtail viremia at least during acute infection^25^. Although peak viral loads were comparable between the two groups, three of the five infected animals in the HVV + SOSIP/3M-052 group had a viral load below the detection limit 10 weeks later (Fig. 2c). This is consistent with the concept that Gag-specific CD8^+^ T cells can lower the viremia set point despite early multiplication of the virus^26^.

### Immune correlates of protection

**Fig. 3:**
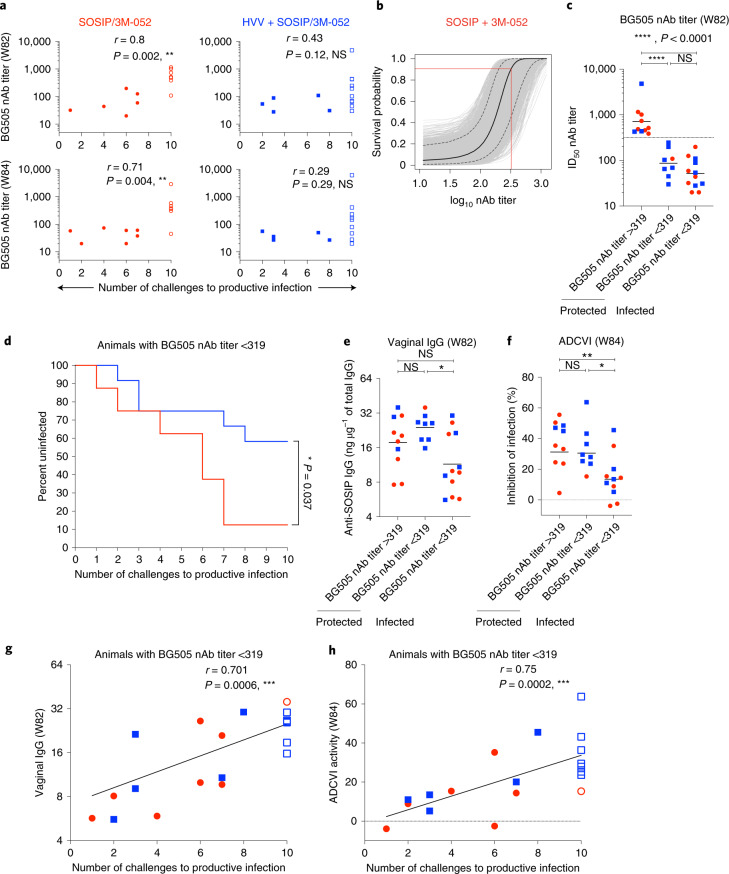
Immune correlates of protection. **a**, Spearman’s correlation between serum autologous nAb ID_50_ titers at 2 weeks before (top) and on (bottom) the day of first challenge (weeks 82 and 84, respectively) and the rate of acquisition of infection in the SOSIP/3M-052 (red circles) and HVV + SOSIP/3M-052 (blue squares) immunization groups, as indicated (*n* = 15 in each group at both time points except *n* = 13 in SOSIP/3M-052 group at week 82). Open symbols indicate animals uninfected at the end of ten challenges. **b**, The probability of survival defined by logistic regression analysis of autologous nAb ID_50_ titers at 2 weeks before the day of first challenge and rate of acquisition of infection in the SOSIP/3M-052 group (*n* = 13). The median probability (black curve) and the 5% and 95% confidence limits (dotted curves) are shown. Red lines extrapolate the neutralization titer (319) associated with 90% survival probability. **c**, Autologous nAb titers in animals (groups indicated by red and blue as in **a**, *n* = 15 in each group) clustered by infection status and nAb cutoff value of 319 identified in **b**. Geometric means are shown. Statistical differences were analyzed using two-tailed Mann–Whitney rank-sum test (NS, *P* = 0.16). **d**, Kaplan–Meier survival curve comparing only the animals with autologous nAb titers <319 (*n* = 7 in SOSIP/3M-052 group and *n* = 12 in HVV + SOSIP/3M-052 group). *P* value (two-tailed) denotes statistically significant difference calculated by log-rank (Mantel–Cox) test. **e**,**f**, Anti-SOSIP vaginal IgG quantity at week 82 and ADCVI activity measured in week 84 serum, respectively, are plotted for animals clustered by infection status and nAb titer (*n* = 15 in each immunization group). The ADCVI data represent the average of two independent assays using two different PBMC donors. Medians are shown. Statistical differences were analyzed using a Mann–Whitney rank-sum test (**P = 0.006, *P < 0.05). **g**,**h**, Spearman’s correlation between the rate of acquisition of infection and anti-SOSIP vaginal IgG at 2 weeks before challenge (week 82) or ADCVI activity on the day of challenge (week 84) in animals with autologous nAb titers <319 (*n* = 7 in SOSIP/3M-052 group and *n* = 12 in HVV + SOSIP/3M-052 group). In all correlation plots, *r* and *P* represent Spearman’s *r* and two-tailed *P* values.

Next, we sought to identify correlates of protection. Consistent with a recent study^11^, we found a significant correlation between serum nAb titers measured 2 weeks before challenge or on the day of challenge (2 and 4 weeks after final vaccination, respectively) and the rate of acquisition of infection in the SOSIP/3M-052 group (Fig. 3a). Notably, there was no such correlation in the HVV + SOSIP/3M-052 group (Fig. 3a). We applied a logistic regression model to define the absolute nAb titer associated with protection in the SOSIP/3M-052 group. The model predicted a 90% probability of non-infection at a peak serum neutralization titer of 319 (2.5 log_10_) (Fig. 3b). Consistent with this prediction, seven of eight protected animals in the SOSIP/3M-052 group had a nAb titer >319. In contrast, every animal below this threshold, except one (nAb titer 58), was infected. We infer that the nAb titer was the primary correlate of protection in animals immunized with SOSIP/3M-052 alone.

**Extended Data Fig. 5:**
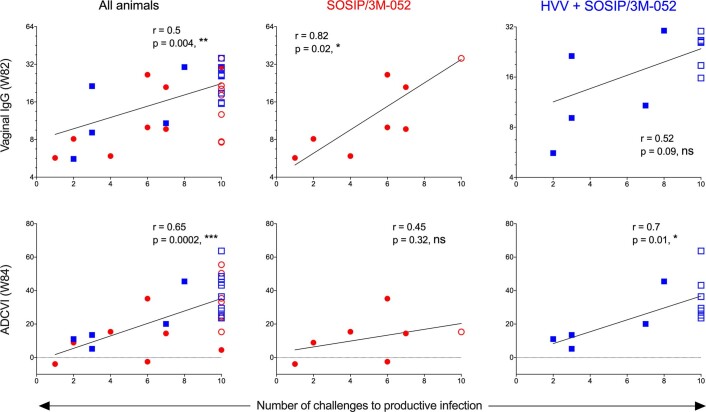
Correlation of vaginal binding IgG and ADCVI responses with protection. Spearman’s correlation between vaginal IgG response at week 82 (top panels) and ADCVI at week 84 (bottom panels) with rate of acquisition of infection. The analysis included all animals (n = 15 per group) from both vaccine groups irrespective of the nAb titers (left panel), animals with nAb titers < 319 in the SOSIP/3M-052 group (middle panel, n = 8) or HVV + SOSIP/3M-052 group (right panel, n = 12). SOSIP/3M-052 and HVV + SOSIP/3M-052 immunization groups are indicated by red circles and blue squares, respectively. Open symbols indicate animals uninfected at the end of 10 challenges. The r and p values represent Spearman’s r and two-tailed p values.

Similarly, in the HVV + SOSIP/3M-052 group, all 3 animals (out of 15) with nAb titers >319 were protected. Notably, however, 7 of the 12 animals (60%) with nAb titers <319 were also protected (Fig. 3c). This striking difference between the two groups was further evident in the Kaplan–Meier analysis of animals with nAb titers <319, which reveals statistically significant protection in the HVV + SOSIP/3M-052 subgroup compared to the corresponding SOSIP/3M-052 subgroup (Fig. 3d). In addition, anti-trimer vaginal IgG-binding antibody and antibody-dependent cell-mediated viral inhibition (ADCVI), a measure of the ability of antibodies to inhibit virus infection in the presence of Fc receptor-expressing monocytes and natural killer (NK) cells, were higher in the protected animals with nAb titers <319, in comparison to the infected animals (Fig. 3e–h and Extended Data Fig. 5). Notably, vaginal IgG and ADCVI responses also correlated with protection in our previous study, in which young animals that received HVV + Env (non-native gp140) immunizations were protected^22^. These results imply that while nAbs represent the primary correlate of protection against HIV, the induction of cellular immune responses may synergize with binding antibodies with effector functions at the site of viral exposure and offer an additional mechanism of protection, effectively lowering the threshold of nAb associated with protection.

**Extended Data Fig. 6:**
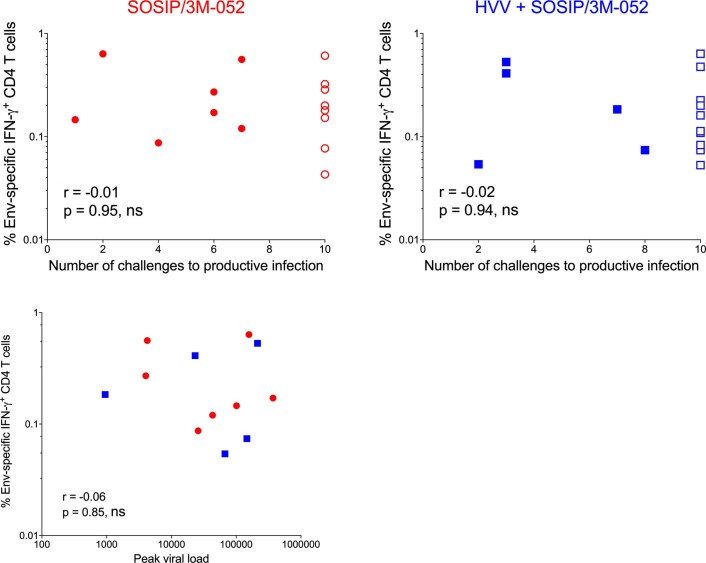
Correlation of Env-specific CD4 T cell responses with protection. Spearman’s correlation between Env-specific CD4 T cell frequencies at week 81 (3 weeks prior to challenge, n = 15) and rate of acquisition of infection **(a)**, and peak viral load, n = 12 infected animals in total **(b)**. SOSIP/3M-052 and HVV + SOSIP/3M-052 immunization groups are indicated by red circles and blue squares, respectively. Open symbols indicate animals uninfected at the end of 10 challenges. The r and p values represent Spearman’s r and two-tailed p values.

**Extended Data Fig. 7:**
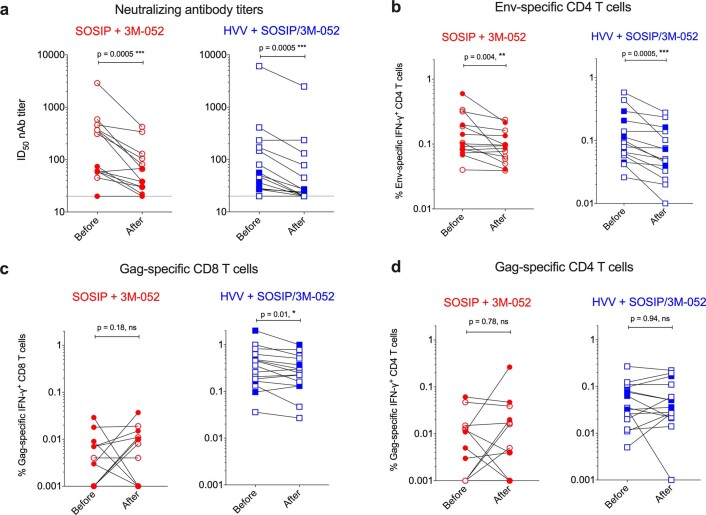
Autologous nAb and T cell responses before and after challenge. **a**, Autologous nAb titers in animals (SOSIP + 3M-052 and HVV + SOSIP/3M-052 groups indicated by red and blue) measured at weeks 84 and 94, the day of the first and last of the ten challenges. For animals infected before week 94, we used sera collected two weeks before the detection of viremia, the predicted actual day of infection. Env-specific CD4 T cell **(b)**; Gag-specific CD8 **(c)** and CD4 T cell **(d)** responses in PBMCs harvested before and after the first 10 challenges. For animals infected before week 94, PBMCs harvested at a time point before actual infection were used. Open and closed symbols indicate animals uninfected or infected after the 10 challenges. Wilcoxon paired test was used for statistical analysis of all panels. All p-values are based on two-tailed tests. N = 15 per group in all the panels.

**Extended Data Fig. 8:**
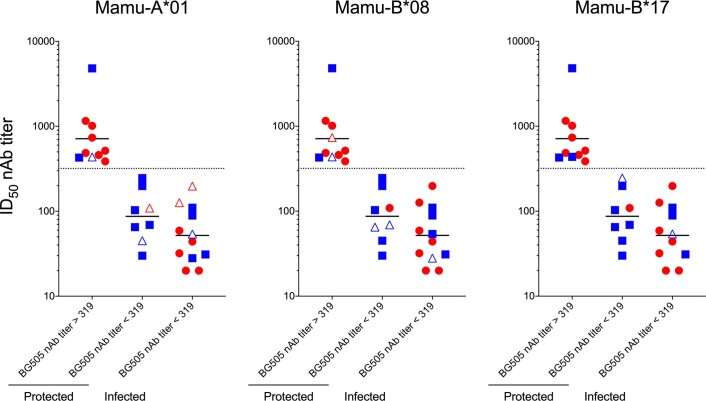
Classification of animals based on the magnitude of autologous nAb titers and infection status. Autologous nAb titers in animals (SOSIP + 3M-052 and HVV + SOSIP/3M-052 groups indicated by red and blue) classified by infection status. The open triangles represent animals expressing restrictive MHC alleles mentioned in the headings. N = 15 per immunization group in all the panels.

Vaccine-specific type 1 helper CD4^+^ T cells have been shown to be associated with reduced vaccine efficacy^27^. However, in these experiments, we did not see a correlation between Env-specific IFN-γ^+^CD4^+^ T cells in blood and protection or viral load (Extended Data Fig. 6). Furthermore, to address whether nAbs or T cell responses were boosted by the challenge virus in a manner that helped durable protection, we compared nAb responses measured at weeks 84 and 94 (or a time point before infection in the case of infected animals), the day of the first and last challenges. The nAb response declined in all animals, irrespective of their immunization group or infection status (Extended Data Fig. 7a). Thus, there was no evidence of enhanced nAb response induced by the challenge virus. Similarly, there was no T cell activation, Gag- or Env-specific CD8^+^ and CD4^+^ T cells, following challenge (Extended Data Fig. 7b–d). We also addressed whether the protective major histocompatibility complex (MHC) class I alleles, Mamu-A*01, Mamu-B*08 and Mamu-B*17 had any role in the enhanced protection observed in the HVV + SOSIP/3M-052 group^28^. Mamu-A*01 animals were equally distributed between the groups and even though there were more animals with Mamu-B*08 and Mamu-B*17 in the HVV + SOSIP/3M-052 group, the numbers of animals with these alleles were too few to draw any conclusions. Nevertheless, there does not seem to be any association between the presence of these alleles and protection (Extended Data Fig. 8).

### In situ T cell reactivation stimulates an antiviral program locally

**Extended Data Fig. 9:**
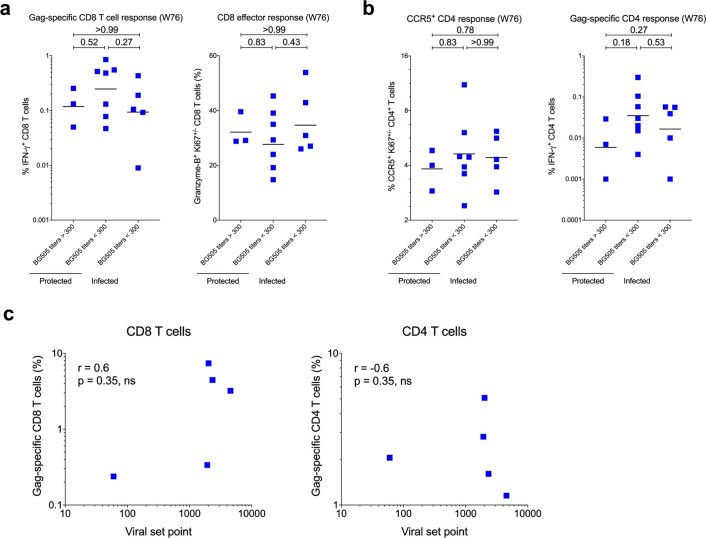
Correlates of protection in the HVV + SOSIP/3M-052 group. **a**, Gag-specific IFN-γ^+^ CD8^+^ T cell and granzyme B^+^ effector CD8^+^ T cell responses in group 2 animals classified by infection status. **b**, Frequencies of Gag-specific IFN-γ^+^ CD4^+^ T cells or CCR5^+^ CD4^+^ T cells at week 76, the available time point closest to the challenge, in animals classified by infection status and nAb titers. N = 15 in panels **a** and **b**. The horizontal lines indicate geometric means. **c**, Spearman’s correlation between Gag-specific IFN-γ^+^ CD8 (left panel) or CD4 (right panel) T cells and viral set point (n = 5 infected animals). The r and p values represent Spearman’s r and two-tailed p values.

The ability of CD8^+^ T cells to control viral replication has been well established but the question of whether they consistently prevent infection is unresolved^13,29–31^. Vaccine-induced CCR5^+^ type 1 helper CD4^+^ T cells are known to be associated with increased simian immunodeficiency virus (SIV) acquisition^32^ and are targets for HIV infection^33,34^. In our study, we did not find a significant difference in Gag-specific T cells in blood between protected versus infected animals with nAb titers <319. Specifically, neither the magnitude of Gag-specific IFN-γ^+^ or granzyme B-producing CD8^+^ T cells (Extended Data Fig. 9a), nor the frequency of CD4^+^CCR5^+^ T cells or Gag-specific IFN-γ producing CD4^+^ T cells differed between these animals (Extended Data Fig. 9b). In addition, the Gag-specific T cell responses did not correlate with the viral set point (Extended Data Fig. 9c).

**Fig. 4:**
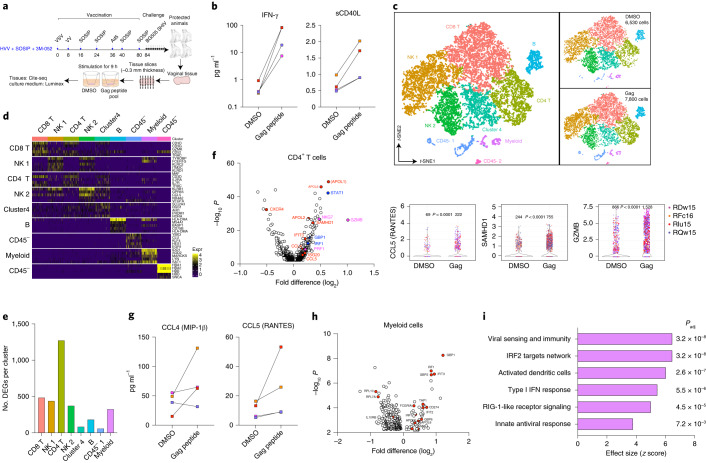
Single-cell RNA-seq analysis of vaginal tissues stimulated ex vivo with Gag peptide pool. **a**, A schematic representation of the analysis of four protected animals in the HVV + SOSIP/3M-052 group. **b**, Concentration of IFN-γ and sCD40L in culture supernatants as measured by Luminex assay. Each symbol represents an individual animal. **c**, Transcriptome-based clustering of 14,571 CITE-seq single-cell expression profiles visualized by *t*-SNE in two-dimensional space. The smaller clusters on the right show distribution of cells from DMSO or Gag peptide pool stimulation conditions. **d**, Heat map reporting scaled and log-transformed expression (Expr) of transcripts; discriminative markers are indicated on the right of each cluster indicated on the left and top. Expression levels are plotted in 100 cells randomly selected per cluster. **e**, Number of significantly induced genes per cluster following stimulation with Gag peptide pool, compared to DMSO control, are plotted. **f**, Volcano plot showing DEGs in the CD4^+^ T cell cluster (*n* = 2,394 cells). Genes highlighted in blue are prototype IFN-induced genes, markers of cytolytic activity are highlighted in magenta and well-known HIV-restriction factors are highlighted in red. Violin plots on the right show number of cells in each condition that express the markers indicated. Statistical differences were analyzed using two-tailed Mann–Whitney rank-sum test. **g**, Concentration of MIP-1β and RANTES in culture supernatants measured using Luminex and ELISA, respectively. **h**, Volcano plot showing DEGs between cells treated with DMSO or the Gag peptide pool in the myeloid cell cluster (*n* = 288 cells). IFN-induced antiviral genes are highlighted in red. **i**, Pathway analysis showing blood transcriptional modules that were significantly upregulated following stimulation with the Gag peptide pool. False discovery rate-corrected *P* values are indicated on the right. All DEGs and pathways in **e**,**f**,**h** and **i** are defined by two-tailed Mann–Whitney rank-sum test with false discovery rate-corrected *P* < 0.05.

Our recent work demonstrates that vaccination with the HVV regimen described in this study induces a high magnitude of CD8^+^ T cells in vaginal tissues (Extended Data Fig. 4)^22^. Given the lack of correlation of Gag-specific CD8^+^ T cells in blood with protection in this study, we hypothesized that CD8^+^ T cells elicited by HVV vaccination might play a local role in enhancing antibody-mediated protection. In this context, a recent study in mice demonstrates that local reactivation of CD4^+^ T cells in vaginal tissues results in enhanced recruitment of memory B cells that secrete high levels of antibody in situ^35^. Furthermore, previous studies in mice have demonstrated that local CD8^+^ T cell reactivation stimulates in situ activation of dendritic cells and NK cells in female reproductive tracts^36,37^. However, the answers as to whether local T cell reactivation can result in an antiviral program, and in particular whether such antiviral responses might induce HIV-restriction elements, are unknown. To address these questions, we isolated vaginal tissues, following necropsy, from four of ten protected macaques (all with nAb titers <319) in the HVV + SOSIP/3M-052 group, at 3 months after the tenth viral challenge. We stimulated vaginal tissues for 9 h with an overlapping SIV-Gag peptide pool to activate T cells in situ and then performed a single-cell RNA-sequence (RNA-seq) analysis on cells isolated from tissues (Fig. 4a).

**Extended Data Fig. 10:**
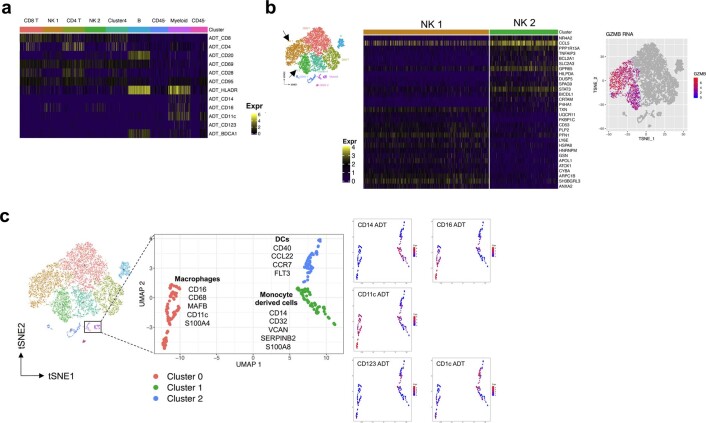
Analysis of single-cell clusters identified by gene expression profiles. **a**, Heat-map reporting scaled and log-transformed expression of ADTs by the cell clusters indicated on the top. Expression levels are plotted in 100 cells randomly selected per cluster. **b**, Heat-map showing expression of genes discriminative of NK cell clusters 1 and 2 (3,099 and 1,688 cells, respectively). **c**, Sub-clustering of myeloid cells (288 cells) by hierarchical clustering of ADT expression. The myeloid cell clusters are visualized by UMAP and the genes discriminative of each cluster is shown. The expression levels of ADT signals are plotted on the right.

The activation of T cells by Gag peptide stimulation in vitro was first evaluated by measuring the secretion of cytokines and chemokines into the culture supernatants. There was significantly enhanced secretion of IFN-γ in each sample (ranging from 10- to 100-fold) following stimulation with the Gag peptide pool relative to DMSO controls (Fig. 4b). Soluble CD40L was also upregulated, albeit by a more modest twofold, perhaps reflecting activation of a smaller subset of Gag-specific CD4^+^ T cells (Fig. 4b). Next, we simultaneously evaluated gene and protein expression of single cells in stimulated and unstimulated vaginal tissues using cellular indexing of transcriptomes and epitopes by sequencing (CITE-seq) method. Transcriptome-based clustering of the resulting 14,571 single-cell gene expression profiles, derived using a graph-based clustering technique described recently^38^, identified nine distinct cell clusters (Fig. 4c). The clusters were visualized in two-dimensional space using *t*-distributed stochastic neighbor embedding (*t*-SNE). The top five genes that defined clusters are shown in Fig. 4d and signals from antibody-derived tags (ADTs) are shown in Extended Data Fig. 10a. As expected, CD8^+^ and CD4^+^ T cells (4,274 and 2,394 cells, respectively) formed two largely non-overlapping clusters and expressed markers such as *CD3D* and *CD3G*. A third cluster (cluster 4) that contained a mixture of CD8- and CD4-expressing cells, distinguished by expression of genes such as *RORA* and *SLC2A3*, did not uniquely express genes typically associated with specific T cell subtypes. Of the remaining five clusters, two involved NK cells (4,787 cells in total), one myeloid cell cluster (288 cells) and two CD45^−^ cell clusters (549 cells). Differential expression of granzyme B distinguished NK cell subsets; NK cell subset 1 expressed higher granzyme B levels than NK cell subset 2. Conversely, NK subset 2 cells were enriched in genes such as STAT3 and GPR65 (Extended Data Fig. 10b). Next, using ADTs (CD14, CD16, CD11c, CD123 and CD1c), we identified three myeloid cell subclusters (Extended Data Fig. 10c). Cluster 0 expressed CD16 and CD11c ADTs and cluster-defining genes included macrophage markers *CD68* and *MAFB*. Clusters 1 and 2 were marked by CD14 and CD1c, respectively. Cluster 1 was further defined by genes including *CD32*, *VCAN*, *SERPINB2* and *S100A8* in addition to CD14, whereas cluster 2 was defined by *CD40*, *CCR7* and *CCL22*.

We then assessed gene expression induced by Gag peptide stimulation. The number of cells in DMSO (6,530 cells) and Gag peptide (7,800 cells) stimulation conditions were comparable (Fig. 4c). The remaining 241 of the total 14,571 cells profiled by CITE-seq could not be reliably assigned to a treatment group. Unbiased differential gene expression analysis identified the highest number of differentially expressed genes (DEGs) in CD4^+^ T cells (Fig. 4e). In addition to induction of a number of IFN-γ-induced genes (GBP family of proteins, *IRFs*, *TRIM5*, *TRIM21*, *OAS2*, *MT2A* and *STAT1*)^39^, granzyme B and cytotoxicity-associated genes (*PRF1, NKG7*) were upregulated, suggesting induction of potentially cytotoxic CD4^+^ T cells. Notably, CD4^+^ T cells showed enhanced levels of well-known HIV-restriction factors such as *CCL5*, *CCL4*, *SAMHD1* and several members of the apolipoprotein family^40–42^ (Fig. 4f). Induction of CCL4 and CCL5 was also found in the culture supernatant as measured by Luminex assay or ELISA (Fig. 4g). Such antiviral factors can render CD4^+^ T cells more resistant to infection^43^. We further examined whether myeloid cells, including dendritic cells and macrophages, showed induction of antiviral genes. We found significant induction of genes such as *IRF1*, *IFIT3*, *GBP1* and *TAP1* (Fig. 4h). Pathway analysis using blood transcriptional modules identified viral sensing and immunity, IRF1 signaling, activated dendritic cells, type I IFN response and innate antiviral response as the top pathways induced by Gag stimulation (Fig. 4i).

### Cellular immune responses plus nAbs provide durable resistance

**Fig. 5:**
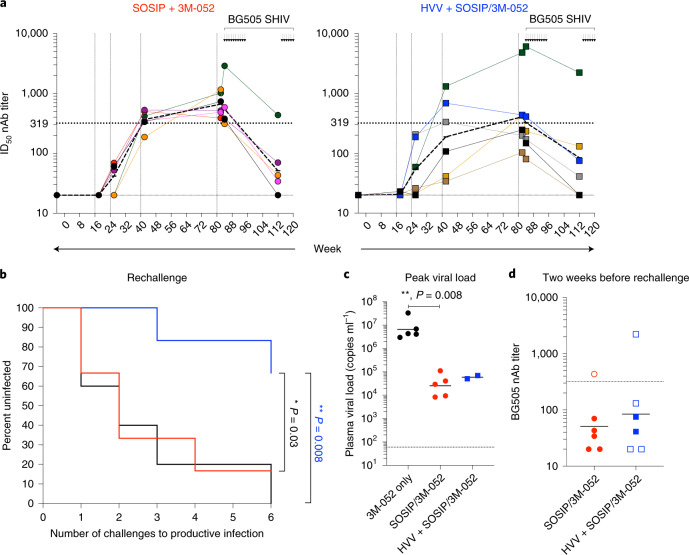
Durability of vaccine-induced protection. **a**, Kinetics of nAb titers in animals re-challenged (second set of six arrows) after surviving initial challenges (first set of ten arrows), for each immunization group. Each color represents an individual animal. The dark discontinuous lines indicate geometric mean titers for each group. **b**, Kaplan–Meier survival curves showing the fraction of uninfected animals following each challenge. Six animals from SOSIP/3M-052 (red) and HVV + SOSIP/3M-052 (blue) groups that survived the initial ten challenges were re-challenged a further six times (see **a**). The black curve indicates five control animals that were challenged six times over the same period. *P* values (two-tailed) denote statistically significant differences calculated by log-rank (Mantel–Cox) test. **c**, The peak plasma viral loads of animals infected during the re-challenge. Geometric means are indicated. Statistical differences were analyzed using a two-tailed Mann–Whitney rank-sum test (**P = 0.008). **d**, Autologous nAb titers measured 2 weeks before re-challenge (at week −112). Closed and open symbols indicate animals that were infected and uninfected, respectively, after the six additional challenges (*n* = 6). Geometric means are indicated by horizontal lines.

Finally, to determine whether protection was durable, six uninfected animals from groups 1 and 2 were re-challenged 5 months after the last of the initial ten challenges (at week −114; Fig. 1a) and hence about a year and a half from the last viral vector immunization and over 8 months from the last protein immunization. The autologous nAb titers in all but two of the surviving animals (one in each group) had, by now, declined considerably below the threshold value of 319 (Fig. 5a). Notably, however, when the immunized animals were re-challenged a further six times with the same SHIV-BG505 virus, there was significantly greater protection in group 2 than in group 1 (66.7% compared to 16.7%; Fig. 5b). Four of five control animals challenged at the same time were infected within three challenges. The peak viral load was curtailed significantly in both vaccinated groups as seen previously (Fig. 5c and Fig. 2b). Of particular note is that the data from the re-challenge validated the serum nAb titer threshold of 319, identified earlier (Fig. 3b). Thus, in the re-challenge study, the one animal in group 1 that had a neutralization titer >319 at 2 weeks before the first re-challenge was the only animal that remained protected after the six additional challenges (Fig. 5d). In contrast, but as seen previously, protection in group 2 was not associated with the threshold nAb titer (Fig. 5d). Together, these results show that the protection in animals immunized with HVV + SOSIP/3M-052 was long lived.

## Discussion

Taken together, these data demonstrate that a BG505 SOSIP.664 trimer-induced tier 2 nAb response can protect against low-dose mucosal SHIV challenge. While a serum nAb titer of >300 is generally required for protection, this threshold was significantly reduced when combined with HVV immunization that induced high levels of CD8^+^ and CD4^+^ T cells in blood and vaginal tissues. Notably, the durability of protection induced in the presence of cellular immunity plus nAbs was significantly higher. The enhanced durability achieved by combining cellular immunity and antibodies will be of interest to vaccinology in general^44^.

Our results further demonstrate that serum nAb titer represents the primary correlate of protection in the SOSIP/3M-052 group, consistent with a recent study^11^. However, in addition, we show that vaginal Env-specific binding antibody responses and ADCVI likely offer auxiliary mechanisms of protection when nAb antibody responses are suboptimal. Notably, these auxiliary mechanisms seem to be effective only in the HVV + SOSIP/3M-052 group, arguing for a synergistic role for Gag-specific T cells. Alternatively, it is possible that in the animals immunized with HVV + SOSIP/3M-052, there were enhanced nAb titers in the vaginal mucosae. We could not measure nAb response in vaginal secretions to formally evaluate this hypothesis owing to limitations in sampling volume. However, because both the HVV + SOSIP/3M-052 and SOSIP/3M-052-alone groups had similar levels of serum nAb and as the serum antibody pool represents the dominant source of mucosal antibodies during parenteral vaccination^23,45^, it seems unlikely that mucosal neutralization titers would differ between the two groups. Nevertheless, this remains to be determined in the future.

Furthermore, using single-cell transcriptional profiling we provide evidence for mucosal T cell-mediated activation of a set of antiviral restriction factors in mucosal innate cells and CD4^+^ T cells and demonstrate that vaccination strategies that induce both nAbs and T cells can confer enhanced and durable protection against HIV acquisition. We hypothesize that the CD8^+^ TRMs induced by the HVV immunization regimen in vaginal tissues provide an additional layer of protection in animals with low nAb titers. Analysis of TRMs in vaginal biopsies before challenge with the virus would provide direct evidence for their role in this regard; however, that was not performed in this study because our primary goal was to assess protection and we wanted to avoid local trauma and inflammation at the site of infection before challenge. Future studies are warranted to evaluate the role of TRMs in protection. Of particular interest is to address whether inclusion of cellular immune responses and nAbs against one strain can protect against heterologous strains of (S)HIV. Such a strategy aimed at harnessing the synergistic potential of antibodies and T cells may also have broader utility in inducing protective immunity against diverse pathogens such as influenza, tuberculosis and malaria.

## Methods

### Animal subjects and experimentation

Forty-five female rhesus macaques (*Macaca mulatta*) of Indian origin, aged 3–15 years were assigned to the study (Supplementary Table 1). All animals were confirmed negative for SIV infection and genotyped for expression of MHC-I alleles, Mamu-A*01, Mamu-B*08 and Mamu-B*17. Animals were randomly distributed between the groups. Age and MHC-I alleles were matched between both vaccine groups. Animals were maintained as per National Institutes of Health (NIH) guidelines and all procedures were approved by the Institutional Animal Care and Use Committee of Emory University. All animals were freshly enrolled for this study and had not been involved in previous experiments.

### Immunogens, vaccinations and challenge

The BG505 SOSIP.664 recombinant Env glycoprotein trimer was produced in CHO cells under current good manufacturing practice conditions as described previously^46^ and was obtained from the International AIDS Vaccine Initiative. The preparation of PLGA nanoparticles encapsulating 3M-052 and the details of recombinant viral vectors expressing Gag (VSV, VV and Ad5) are described elsewhere^22^. VSV-Gag is an attenuated, replication-competent, live VSV-New Jersey serotype virus prepared as described elsewhere^47^ using SIVmac239 Gag as the insert. The virus replicates in macaques, but previous attempts to recover virus from immunized animals have been unsuccessful, suggesting rapid control by the immune system^48^. VV-Gag is of the WR strain in which the thymidine kinase gene was rendered inactive by the insertion of SIVmac239 Gag. Recombinant thymidine kinase-negative VV is live-attenuated and less pathogenic compared to the wild-type strain^49^. Recombinant Ad5-Gag is a replication-incompetent viral vector in which the viral genes, E1 and E3, were deleted^50^.

SOSIP/3M-052 vaccinations were administered subcutaneously as split doses in hind limbs. Each immunization consisted of two doses of 100 µg BG505 SOSIP.664 and 37.5 µg 3M-052 (encapsulated in PLGA) in 0.5 ml of sterile phosphate-buffered saline (PBS). VSV-Gag, VV-Gag and Ad5-Gag were administered intravenously at doses of 5 × 10^7^, 1 × 10^7^ and 7 × 10^10^ plaque-forming units, respectively, in 1 ml of PBS. Intravenous administration of viral vectors was aimed at expanding the number of effector memory T cells and TRMs as seen in mice previously^21,22^. For group 3, immunization consisted of two doses of 37.5 µg of 3M-052 in PLGA, but without BG505 SOSIP.664 trimer, in PBS as described above.

Animals were atraumatically challenged weekly via an intravaginal route with SHIV-BG505.332N.375Y∆CT virus, produced in rhesus CD4^+^ T cells, as described previously^24^. SHIV-BG505.332N.375Y∆CT contains a *vpu-env-gp140* segment of the transmitter/founder HIV-1 subtype-A virus, BG505, in a SIVmac766 backbone. The Env gene has S375Y mutation that allows enhanced binding and replication in rhesus CD4^+^ T cells. The virus replicated persistently in macaques at titers comparable to HIV-1 in humans and elicited nAb responses as seen in HIV-1 infections^24^. The challenge virus stock was titrated and used at a 1:3 dilution in a total volume of 1 ml serum-free RPMI 1640 medium, which is equivalent to 3.5 × 10^8^ virions or 50 ng p27, per challenge. Animals were considered infected if their plasma SHIV viral RNA loads were >60 copies ml^−1^ for 2 weeks consecutively. Once they were infected, they were not further challenged. The ‘number of challenges to productive infection’ was defined as the time point 2 weeks before the first detection of viremia (>60 SHIV RNA copies ml^−1^).

### Measurement of serum-binding IgG

ELISAs to detect anti-trimer antibodies were performed as described previously^7^, with minor modifications. His-tagged (-GSGSGGSGHHHHHHHH) BG505 SOSIP.664 trimers were coated onto Ni-NTA HisSorb plates (Qiagen) by addition at 1.5 µg ml^−1^ in TBS (150 mM NaCl, 20 mM Tris) for 2 h at room temperature. Unbound trimers were removed by three washes with TBS before the wells were blocked with TBS plus 2% nonfat milk powder (TBS-M) for 30 min. After three more washes with TBS, serial dilutions of macaque sera (or, as a positive control, 2G12 broadly neutralizing antibody) were added in TBS-M plus 20% sheep serum for 2 h, followed by three more TBS washes. Horseradish peroxidase-labeled goat anti-rhesus immunoglobulin G (IgG) (H + L) (Southern Biotech) was added for 1 h at a 1∶3,000 dilution in TBS-M, followed by five washes with PBS (137 mM NaCl, 2.7 mM KCl, 10 mM Na_2_HPO_4_, 1.8 mM KH_2_PO_4_ (pH 7.4)) plus 0.05% Tween-20. Colorimetric detection was performed using the 1-Step Ultra TMB-ELISA substrate (Thermo Fisher Scientific).

### Measurement of serum IgA and mucosal antibodies

Concentrations of anti-trimer IgA in serum and IgA and IgG antibodies in secretions collected with Weck-Cel sponges^51^ were measured by binding antibody multiplex assay using BG505 SOSIP.664 gp140 that had been crosslinked via amide bonds to carboxylated magnetic BioRad BioPlexPro beads as described^52,53^. Briefly, protein-bound beads were mixed overnight at 1,100 r.p.m. and 4 ˚C, with tenfold dilutions of sample or standard, then developed with biotinylated goat anti-monkey IgA or IgG antibody (Rockland Immunochemicals) and neutravidin-phycoerythrin (Southern Biotech). Concentrations of anti-SOSIP antibody in each sample were normalized by dividing by the total IgA or IgG concentration, measured by ELISA using plates coated with goat anti-monkey IgA or IgG (Alpha Diagnostics) and the above biotinylated antibodies. The standard in both anti-trimer and total IgA assays was the anti-CD4 binding site rhesus b12 dimeric IgA monoclonal antibody (NHP Reagent Resource). The standard in anti-trimer and total IgG assays was IgG purified from sera of SHIV-infected macaques and a pool of normal rhesus macaque sera, respectively.

### Neutralizing antibody assay by the Emory Vaccine Center

The HIV-1 Env clone BG505.W6M.Env.C2 (GenBank accession code DQ208458) that had been modified to contain the N332 glycan (T332N)^9^ was used to measure autologous neutralization for the majority of experiments. The SHIV.BG505.332N.375Y virus stock, prepared by transfection into HEK293T cells, provided by G. Shaw was used directly to assess autologous neutralization. The TZM-bl neutralization assay used has been described previously^54–66^. Briefly, BG505.T332N Env pseudovirus was prepared by transfecting the Env-expressing plasmid DNA alongside HIV-1 SG3ΔEnv proviral backbone DNA into 293 T cells. Pseudovirus stock was collected from the HEK293T cell supernatant 48–72 h after transfection, clarified by centrifugation, aliquoted into small volumes and frozen at −80 °C. Env pseudovirus (2,000 infectious units in DMEM with ∼3.5% (vol/vol) FBS (Hyclone) and 40 µg ml^−1^ DEAE-dextran) was mixed with fivefold serial dilutions of heat-inactivated serum and added to plated TZM-bl cells. At 48 h after infection, the cells were lysed and luciferase activity was measured using a BioTek Cytation 3 or Synergy Neo2S multimode reader. The average background luminescence from a series of uninfected wells was subtracted from each experimental well. Each experimental well was compared against a well without a test reagent to establish 100% infectivity. All assays utilized duplicate wells and were repeated at least once independently. Neutralization ID_50_ titer values were calculated in GraphPad Prism using the dose–response inhibition analysis function with variable slope, log-transformed *x* values and normalized *y* values.

### Neutralizing antibody assay by the Duke Central Immunology Laboratory

Neutralizing antibody activity was measured in 96-well culture plates by using Tat-regulated luciferase reporter gene expression to quantify reductions in virus infection in TZM-bl cells. Assays were performed essentially as previously described^67^ using BG505.T332N Env pseudovirus produced in HEK293T cells. Serum samples were heat-inactivated at 56 °C for 1 h before assay. They were diluted over a range of 1:20 to 1:43,740 in cell culture medium and preincubated with virus (~150,000 relative light unit equivalents) for 1 h at 37 °C before addition of cells. Following a 48 h incubation, cells were lysed and luciferase activity was determined using a microtiter plate luminometer and BriteLite Plus Reagent (Perkin Elmer). Neutralization titers are the sample dilution (for test samples) at which relative luminescence units (RLUs) were reduced by 50% compared to RLUs in virus control wells after subtraction of background RLUs in cell control wells.

### T cell assays

The frequency of Gag-specific CD8^+^ T cells was enumerated using tetramer staining. PBMCs isolated from fresh blood collected in cell preparation tubes were stained with a viability dye (fixable viability dye, eBioscience) in PBS followed by staining with a cocktail containing antibodies to CD4 (clone OKT4, BioLegend), CD28 (clone CD28.2, BioLegend), CD127 (clone eBioRDR5, eBioscience), CCR7 (clone G043H7, BioLegend), CXCR3 (clone G025H7, BioLegend), CD95 (clone DX2, BioLegend), CD45Ra (clone MEM-56, Invitrogen), CD8a (clone RPA-T8, BioLegend) and CD3 (clone SP34–2, BD Biosciences) and the Mamu-A*01 MHC-I tetramer specific for SIVmac239 Gag immunodominant peptide CM9 (181-CTPYDINQM-189) conjugated to allophycocyanin in PBS containing 5% FBS (Corning Life Sciences). Cells were then fixed and permeabilized using the fix/perm and perm/wash buffer (BD Biosciences) before intracellular staining with Ki67 (clone B56, BD Biosciences) and granzyme B (clone GB12, Invitrogen). The samples were analyzed using a BD LSR-II flow cytometer (BD Biosciences).

Functional responses of Gag- and Env-specific CD8^+^ and CD4^+^ T cells were measured using intracellular cytokine staining (ICS) assay. One to two million PBMCs were incubated in 200 μl of RPMI 1640 medium containing 10% FBS with anti-CD28 (1 μg ml^−1^, clone CD28.2), anti-CD49d (1 μg ml^−1^, clone 9F10) and different stimulation conditions as follows: (1) DMSO as a negative control; (2) Gag peptide pool (1–125 peptides derived from SIVmac239, cat. no. 6204, NIH AIDS reagent program), consensus subtype-A Env peptide pool (1–110 peptides that induced detectable T cell responses consistently, cat. no. 12734, NIH AIDS reagent program) at a concentration of 1 μg ml^−1^; and (3) phorbol myristate acetate and ionomycin for positive control. Anti-human CD107a (clone H4A3, BioLegend) was added to the culture. Brefeldin A (10 μg ml^−1^) was added after 2 h of incubation and cells were incubated for an additional 4 h. Cells were transferred to 4 °C overnight and stained the next day for expression of cytokines. Cells were washed once with PBS and stained with a viability dye followed by staining with a surface antibody cocktail containing antibodies to CD8 and CD4 (clones same as above). Cells were then washed, fixed and permeabilized with cytofix/cytoperm buffer for 10 min. Permeabilized cells were stained with ICS antibodies to IFN-γ (clone 4S.B3, BioLegend), tumor necrosis factor-α (clone Mab11, eBioscience), IL-2 (clone MQ1-17H12, BioLegend), IL-4 (clone 8D4-8, BioLegend), CD40L (clone 24-31, BioLegend) and CD3. Cells were then washed twice with perm/wash buffer and once with staining buffer before analysis using a BD LSR-II flow cytometer. All flow cytometry data were analyzed using FlowJo software v.10 (TreeStar).

### Analysis of TRM responses in tissues

Peripheral blood lymphocytes were isolated from whole blood and stained as described previously^22^. Single-cell suspensions of splenocytes were made by manual digestion as described elsewhere^68^ followed by lymphocyte isolation with Ficoll Paque Plus gradient (GE Healthcare). Lamina propria lymphocyte single-cell suspensions were isolated from fresh female reproductive tract tissue with collagenase IV^68^. Single-cell suspensions were prepared and stained with Gag-CM9 tetramer and a cocktail of antibodies mentioned in T cell assays and an additional antibody to stain CD69 (clone FN50, BioLegend). ICS assay was also performed as described in T cell assays with the following modifications. The stimulants were no peptide or Gag-CM9 peptide at a concentration of 1 µg ml^−1^ in complete RPMI medium with 10 µg ml^−1^ brefeldin A for 16–18 h. Following stimulation, the cells were stained and analyzed as above. The ICS antibody cocktail included MIP-1β clone D21-1351, BD Biosciences). In situ tetramer staining and immunohistochemistry was performed on tissue sections as described previously^22^.

### Viral load assay

Plasma SHIV RNA copy numbers were determined using a quantitative real-time PCR assay with a sensitivity of 60 RNA copies ml^−1^ using primers directed against SIV *gag* as previously described^69^. Briefly, RNA was extracted from plasma of challenged animals and amplified for SIV *gag* gene using quantitative real-time PCR (Applied Biosystems 7500). All PCR assays were performed on the same day of or the day after sample collection. All samples were assayed in duplicate and the mean values were reported.

### ADCVI assay

ADCVI assay was performed as described previously^70^. Briefly, cryopreserved human PBMCs that had been rested overnight in complete RPMI medium at 37 °C, 5% CO_2_, were incubated in quadruplicate with a 1:100 dilution of heat-inactivated serum from vaccinated animals (week 84 time point) and BG505 SHIV-infected CCR5^+^CEM-NK^r^ cells for 4 d. Cells were then washed to remove any anti-Gag antibodies. After an additional 3-d incubation period, the amount of virus released into the culture medium was measured by p27 capture ELISA. Inhibition of infection was calculated by dividing the amount of p27 in test wells by the average p27 quantity measured for wells in which pooled sera from the adjuvant controls (group 3) were added. The experiment was repeated using a different PBMC donor and results were averaged.

### Ex vivo Transwell tissue culture

Vaginal tissues collected from animals undergoing necropsy were shipped overnight in cold RPMI medium supplemented with 10% FBS and antibiotics. Transwell cultures were set up as described elsewhere^71^. Tissues were sliced into thin sections manually using surgical scalpels. Tissue slices were placed in prewarmed RPMI medium containing 10% FBS, antibiotics, l-glutamine, sodium pyruvate and 2-mercaptoethanol in 12-well plates containing 0.4-µm pore size polycarbonate inserts (Corning). Tissues were stimulated with overlapping SIVmac239 Gag peptide pool (NIH AIDS reagent program) at a concentration of 1 µg ml^−1^ of each peptide or DMSO as a negative control. The total volume of medium per well was 1.3 ml. Tissues were incubated at 37 °C, 5% CO_2_ (normal cell culture incubators) for 9 h. After incubation, culture supernatants were stored at −80 °C for Luminex and ELISA assays. Tissues were processed for CITE-seq analysis.

### Luminex assay

Nonhuman primate 37-plex cytokine/chemokine/growth factor ProcartaPlex panel (cat. no. EPX370-40045-901, Invitrogen) was purchased and used according to the manufacturer’s recommendations. Briefly, beads were added to a 96-well plate and washed in Biotek ELx405 washer. Samples were added to the plate containing mixed antibody-linked beads and incubated at room temperature for 1 h followed by overnight incubation at 4 °C with shaking. Following overnight incubation, plates were washed and biotinylated detection antibody was added and incubated for 75 min at room temperature with shaking. The plate was washed and streptavidin-PE was added. After incubation for 30 min at room temperature, the plate was washed and reading buffer was added to the wells. Each sample was measured in duplicate. Plates were read using a Luminex 200 instrument with a lower bound of 50 beads per sample per cytokine. Custom assay control beads by Radix Biosolutions were added to all wells.

### CCL5 ELISA

CCL5 in culture supernatants was measured using a luminescence-based ELISA protocol. In short, anti-human/primate CCL5 capture antibody (R&D systems) was coated onto hi-bind white opaque ELISA plates (Pierce). The plate was blocked and 100 µl of culture supernatants or serially diluted recombinant CCL5 (R&D Systems) was added and incubated for 2 h at room temperature. After incubation, the wells were washed and biotinylated anti-CCL5 detection antibody (R&D systems) was added and incubated at room temperature for 1 h. The wells were washed again and incubated with Streptavidin-Lucia reagent (Invivogen) at a dilution of 1:1,000. The plates were washed and 50 µl of QUANTI-Luc (Invivogen) reagent was added to each well and luminescence was measured using a Spectramax i3X reader in luminescence mode (Molecular Devices).

### CITE-seq single-cell RNA-seq

Vaginal tissue slices collected after stimulation with DMSO or Gag peptide pool were minced and digested in 1 mg ml^−1^ collagenase type IV. They were then dissociated using a gentleMACS dissociator (Milteyni Biotec) with the settings to dissociate mouse spleen. The dissociation protocol was run three times and samples were filtered through 100-µm strainers. Cells were washed twice with HBSS with 2% FBS. Single-cell suspensions were stained with fixable viability dye in PBS followed by staining with a cocktail of oligonucleotide-labeled antibodies (shown in Supplementary Table 2) and FITC anti-CD45 (clone D058–1283) and PE-CF594 anti-CD3 antibodies. Live single cells were sorted to obtain high viability required for the preparation of single-cell gene expression libraries. As T cells form the major proportion of vaginal tissue lymphocytes, we used CD45 and CD3 antibodies to sort cells into three populations (CD45^+^CD3^+^ T cells; CD45^+^CD3^−^ other leukocytes; and CD45^−^ non-leukocytes). On the basis of cell counts sorted for each population, we mixed a known number of each cell type to enrich for leukocytes other than T cells and non-leukocytes in the single-cell RNA preparation. Mixed cell populations were adjusted to 700 cells per µl in 0.6 ml of DNA LoBind microcentrifuge tubes and 3,000–6,000 cells were loaded onto the chromium controller for single-cell partitioning and RNA preparation (10X Genomics).

Single-cell sequencing libraries were prepared according to manufacturer’s protocols using chromium single-cell 3' reagent kits v.3 (10X Genomics) and modified according to the CITE-seq protocol^72^ to accommodate the addition of ADTs. The cDNA was amplified for ten cycles. Then, cDNA fractions containing ADT or gene expression libraries were separated on the basis of fragment size using 0.6× SPRIselect (Beckman Coulter). ADTs were further purified using 2.0× SPRIselect and amplified by an additional 12 cycles using primers that add P5 and P7 sequences for Illumina sequencing. Generated ADT libraries were then purified using 1.6× SPRIselect. Gene expression libraries and ADT libraries were quality controlled using fragment analyzer (Agilent) and combined at a ratio of 10:1 for sequencing. Pooled libraries were quantified using Qubit (Thermo Fisher Scientific) and sequenced on HiSeq4000 sequencing system (Illumina) at the Stanford Genome Sequencing Service Center. Paired-end sequencing was performed with read lengths of 2 × 101 bp and both indices.

### Single-cell RNA-seq analysis

Sequencing output was processed with Cellranger v.3.0.2 (10X Genomics). Briefly, the output bcl2 file was converted to FASTQ format using mkfastq. The FASTQ files were then aligned with default settings with Cellranger-count, resulting in a transcript and ADT read-count matrix for each sample. Cells were filtered out if they expressed <100 genes or had a mitochondrial gene content >4 s.d. above the median.

Post-processing of raw counts was performed using the Seurat package v.3.0.0 in R 3.5.1. Gene and ADT counts from each sample were merged into a single Seurat object. Counts were log-transformed after normalization by the total counts for that cell and multiplied by 10,000. Principle components (PCs) were then calculated for the dataset using Seurat. Clusters were identified with the Seurat function FindNeighbors with the first seven PC dimensions followed by FindClusters with a resolution of 0.2. To visualize all cells in two-dimensional space, *t*-SNE was performed with the first seven PCs. Cluster-defining genes were determined using the Seurat function FindAllMarkers with default parameters. The top cluster-defining genes were used as markers alongside normalized ADT reads to build a signature gene and surface-protein expression matrix.

Each cluster was then annotated based on the presence of known marker genes in the resulting set of cluster-defining genes. Differential gene expression analysis between Gag peptide-stimulated and control (DMSO) cells in each cluster was performed with a Mann–Whitney rank-sum test. All *P* values were adjusted for multiple hypothesis testing with the Benjamini–Hochberg procedure. Subclustering was performed on innate immune cells using Seurat with the same parameters as for all cells. Individual subclusters of innate cells were visualized with uniform manifold approximation and projection with the uwot package v.0.1.3.

To identify differentially regulated pathways between Gag and DMSO-treated cells within innate cells, MAST^73^ was used to fit a linear mixed model to adjust for gene detection rate. The resulting model was used to perform gene-set enrichment analysis with blood transcriptional gene modules^74^.

### Statistical analysis

The difference between any two groups at a time point was measured using a two-tailed nonparametric Mann–Whitney unpaired rank-sum test. The difference between time points within a group was measured using a Wilcoxon matched-pairs signed-rank test. The statistical differences in Kaplan–Meier survival curves were analyzed by a log-rank test. All correlations were Spearman’s correlations based on ranks. All the analyses were performed with GraphPad Prism v.8.2.0. The statistical tests for CITE-seq analysis were performed using the stats package v.3.5.1 in R.

Bayes generalized linear model (GLM) was used to model the probability of infection as a function of neutralizing antibody titers 2 weeks before challenge. The discrete probability of infection was connected to continuous neutralizing antibody titers using the logit link function and the infection probability was computed for each animal through Bernoulli distribution. GLM was formulated as:

$$\mathrm {logit}(y_i) = ax_i + b;\mathrm {and}\,p_i(x_i,Y_i|a,b) = \mathrm {Bernoulli}(Y_i,y_i)$$

*Y*_*i*_ and *y*_*i*_ are the actual outcome and expected probability of *i*th animal being uninfected, *x*_*i*_ is the *z* score of the neutralizing antibody titer of *i*th animal at week 82, *a* and *b* are regression coefficients and *p*_*i*_ is the probability of actual outcome, *Y*_*i*_. We solved the above GLM formulation through Bayesian inferences as:

$$P\left( {a,b|X,Y} \right) = \frac{{P(X,Y|a,b) \times P(a,b)}}{{P\left( {X,Y} \right)}};\mathrm {likelihood:}\,P(X,Y|a,b) = \mathop {\prod }\limits_{i = 1}^m p_i(x_i,Y_i|a,b)$$

where, $X = (x_1,x_2, \ldots \ldots ,x_m)$, $Y = (Y_1,Y_2, \ldots \ldots ,Y_m)$ and *m* is the total number of animals. The normal distribution, *N* (0,10), was used as previous distribution of *a* and *b*. Metropolis–Hastings algorithm was used to perform a Markov chain Monte Carlo simulation to estimate the posterior distribution of the model parameters, *a* and *b*.

The calculated values for each parameter were: *a* = 5.27, *b* = 0.55 for SOSIP/3M-052 and *a* = 3.69, *b* = 0.48 for HVV + SOSIP/3M-052.

### Reporting Summary

Further information on research design is available in the Nature Research Reporting Summary linked to this article.

## Online content

Any methods, additional references, Nature Research reporting summaries, source data, extended data, supplementary information, acknowledgments, peer review information; details of author contributions and competing interests; and statements of data and code availability are available at 10.1038/s41591-020-0858-8.

## Supplementary information

Supplementary Information Supplementary Tables 1 and 2

Reporting Summary

## Data Availability

Single-cell RNA-seq data are available in the NCBI Gene Expression Omnibus (GSETBD) under accession number GSE138156. Codes used to analyze data and generate figures are available on Github (https://github.com/scottmk777/Rhesus_CITE-seq).

## Code availability

Codes used to analyze data and generate figures are available on Github (https://github.com/scottmk777/Rhesus_CITE-seq).
