# Supplementary material for: T cell-inducing vaccine durably prevents mucosal SHIV infection even with lower neutralizing antibody titers
Source: Nat Med. 2020 May 11;26(6):932–40. doi: 10.1038/s41591-020-0858-8 (PMC7303014; doi:10.1038/s41591-020-0858-8)
Supplement: Supplementary file 1 — Supplementary Tables 1 and 2 [file 41591_2020_858_MOESM1_ESM.pdf]

nature medicine

SUPPLEMENTARY INFORMATION

https://doi.org/10.1038/s41591-020-0858-8

In the format provided by the authors and unedited.

T cell-inducing vaccine durably prevents mucosal SHIV infection even with lower neutralizing antibody titers

Prabhu S. Arunachalam[1,19], Tysheena P. Charles[2,19], Vineet Joag[3,19], Venkata S. Bollimpelli[4,19], Madeleine K. D. Scott[1,5], Florian Wimmers[1], Samantha L. Burton[2], Celia C. Labranche[6], Caroline Petitdemange[4,17], Sailaja Gangadhara[4], Tiffany M. Styles[4], Clare F. Quarnstrom[3], Korey A. Walter[7], Thomas J. Ketas[8], Traci Legere[4], Pradeep Babu Jagadeesh Reddy[4,18], Sudhir Pai Kasturi[2,4], Anthony Tsai[9], Bertrand Z. Yeung[9], Shakti Gupta[10], Mark Tomai[11], John Vasilakos[12], George M. Shaw[13], Chil-Yong Kang[14], John P. Moore[8], Shankar Subramaniam[10], Purvesh Khatri[1,5], David Montefiori[6], Pamela A. Kozlowski[7], Cynthia A. Derdeyn[2] ✉, Eric Hunter[2] ✉, David Masopust[3] ✉, Rama R. Amara[4] ✉ and Bali Pulendran[1,15,16] ✉

[1]Institute for Immunity, Transplantation and Infection, Stanford University School of Medicine, Stanford University, Stanford, CA, USA. [2]Department of Pathology and Laboratory Medicine, Emory Vaccine Center, Yerkes National Primate Research Center, Atlanta, GA, USA. [3]Department of Microbiology and Immunology, Center for Immunology, University of Minnesota, Minneapolis, MN, USA. [4]Department of Microbiology and Immunology, Emory Vaccine Center, Yerkes National Primate Research Center at Emory University, Atlanta, GA, USA. [5]Center for Biomedical Informatics, Department of Medicine, Stanford University, Stanford, CA, USA. [6]Department of Surgery, Duke University School of Medicine, Durham, NC, USA. [7]Department of Microbiology, Immunology, and Parasitology, Louisiana State University Health Sciences Center, New Orleans, LA, USA. [8]Department of Microbiology and Immunology, Weill Medical College of Cornell University, New York, NY, USA. [9]BioLegend, San Diego, CA, USA. [10]Department of Bioengineering, University of California, San Diego, La Jolla, CA, USA. [11]3M Corporate Research and Materials Lab, Saint Paul, MN, USA. [12]3M Drug Delivery Systems, Saint Paul, MN, USA. [13]Department of Medicine, University of Pennsylvania, Philadelphia, PA, USA. [14]Department of Microbiology and Immunology, Schulich School of Medicine & Dentistry, The University of Western Ontario, London, Ontario, Canada. [15]Department of Pathology, Stanford University School of Medicine, Stanford University, Stanford, CA, USA. [16]Department of Microbiology and Immunology, Stanford University School of Medicine, Stanford University, Stanford, CA, USA. [17]Present address: HIV Inflammation and Persistence Unit, Institut Pasteur, Paris, France. [18]Present address: Pfizer, Andover, MA, USA. [19]These authors contributed equally: Prabhu S. Arunachalam, Tysheena P. Charles, Vineet Joag, Venkata S. Bollimpelli. ✉e-mail: cderdey@emory.edu; ehunte4@emory.edu; masopust@umn.edu; ramara@emory.edu; bpulend@stanford.edu

Supplementary Table 1. Animal assignments, MHC-I alleles and age

Group	Animal code	MHC-I allele			Age	Infection status after the first ten challenges
		A*01	B*08	B*17		
SOSIP/3M-052	275_12	-	+	-	6	Uninfected
	RBi16	-	-	-	4	Infected
	RBw15	-	-	-	5	Infected
	RCs15	-	-	-	5	Infected
	RDr15	+	-	-	5	Infected
	REf15	-	-	-	6	Uninfected
	RHe16	-	-	-	5	Uninfected
	RIr15	-	-	-	5	Uninfected
	RLk15	-	-	-	6	Uninfected
	RSf16	-	-	-	5	Uninfected
	RUg16	+	-	-	5	Uninfected
	RUv16	-	-	-	4	Infected
	RYk15	-	-	-	6	Infected
	RYy15	-	-	-	5	Uninfected
	RZg16	+	-	-	5	Infected
HVV + SOSIP/3M-052	131_12	-	-	+	6	Uninfected
	RAb16	+	+	-	5	Uninfected
	RBr15	-	-	-	5	Uninfected
	RDq15	-	-	-	6	Infected
	RDt16	-	+	-	4	Infected
	RDw15	-	+	-	5	Uninfected
	RFc16	+	-	-	5	Uninfected
	RGr15	-	-	-	5	Infected
	RIu15	-	-	-	5	Uninfected
	ROz15	-	-	-	5	Infected
	RPc16	-	-	-	5	Uninfected
	RPe16	+	-	+	5	Infected
	RQw15	-	+	-	5	Uninfected
	RUp16	-	-	-	4	Uninfected
	RYs15	-	-	-	5	Uninfected
3M-052 only	11_16	-	-	-	7	Infected
	13D096	-	-	-	5	Infected
	4_233	-	-	-	14	Infected
	5_107	+	-	-	13	Infected
	RAk17	+	-	-	3	Infected
	RBc17	+	-	-	3	Infected
	REl17	+	-	-	3	Infected
	RGh17	-	-	-	3	Infected
	RJf17	+	-	-	3	Infected
	ROe17	-	-	-	3	Infected
	RPd17	-	-	-	3	Infected
	RVb17	-	-	-	3	Infected
	RYd17	-	-	-	3	Infected
	RZa17	-	-	-	3	Infected
	RZs9	-	-	-	15	Uninfected

Supplementary Table 2. Antibodies used in the CITE-seq analysis

Marker	Clone	Conjugate	Catalogue number
CD4	OKT-4	Totalseq-A0922	Custom conjugate
CD8	RPA-T8	Totalseq-A0080	301067
CD20	2H7	Totalseq-A0100	302359
HLA-DR	L243	Totalseq-A0159	307659
CD14	M5E2	Totalseq-A0081	301855
CD16	3G8	Totalseq-A0083	302061
CD11c	S-HCL-3	Totalseq-A0053	371519
CD123	6H6	Totalseq-A0064	306037
CD1c	L161	Totalseq-A0160	331539
CD69	FN50	Totalseq-A0146	310947
CD28	CD28.2	Totalseq-A0386	302955
CD95	DX2	Totalseq-A0156	305649
