# Supplementary material for: T cell-inducing vaccine durably prevents mucosal SHIV infection even with lower neutralizing antibody titers
Source: Nat Med. 2020 May 11;26(6):932–40. doi: 10.1038/s41591-020-0858-8 (PMC7303014; doi:10.1038/s41591-020-0858-8)
Supplement: Supplementary file 2 — Reporting Summary [file 41591_2020_858_MOESM2_ESM.pdf]

nature research

Corresponding author(s): Bali Pulendran

Last updated by author(s): Mar 10, 2020

Reporting Summary

Nature Research wishes to improve the reproducibility of the work that we publish. This form provides structure for consistency and transparency in reporting. For further information on Nature Research policies, see Authors & Referees and the Editorial Policy Checklist.

Statistics

For all statistical analyses, confirm that the following items are present in the figure legend, table legend, main text, or Methods section.

n/a	Confirmed	
☐	☒	The exact sample size (*n*) for each experimental group/condition, given as a discrete number and unit of measurement
☐	☒	A statement on whether measurements were taken from distinct samples or whether the same sample was measured repeatedly
☐	☒	The statistical test(s) used AND whether they are one- or two-sided<br>*Only common tests should be described solely by name; describe more complex techniques in the Methods section.*
☐	☒	A description of all covariates tested
☐	☒	A description of any assumptions or corrections, such as tests of normality and adjustment for multiple comparisons
☐	☒	A full description of the statistical parameters including central tendency (e.g. means) or other basic estimates (e.g. regression coefficient) AND variation (e.g. standard deviation) or associated estimates of uncertainty (e.g. confidence intervals)
☐	☒	For null hypothesis testing, the test statistic (e.g. *F*, *t*, *r*) with confidence intervals, effect sizes, degrees of freedom and *P* value noted<br>*Give P values as exact values whenever suitable.*
☐	☒	For Bayesian analysis, information on the choice of priors and Markov chain Monte Carlo settings
☐	☒	For hierarchical and complex designs, identification of the appropriate level for tests and full reporting of outcomes
☐	☒	Estimates of effect sizes (e.g. Cohen's *d*, Pearson's *r*), indicating how they were calculated

Our web collection on statistics for biologists contains articles on many of the points above.

Software and code

Policy information about availability of computer code

Data collection	Flow cytometry data were collected using BD FACSDiva v.8.01 software associated with the BD LSR-II flow cytometer.<br>CITE-seq single-cell RNA sequencing data were processed with CellRanger v3.0.2 from 10X-genomics to obtain bcl2 files for alignment.
Data analysis	Flow cytometry data were analyzed using FlowJo software v10.0 (TreeStar Inc.).<br>CITE-seq single-cell RNA sequencing data were analyzed in R v3.5.1. Packages used: Seurat version 3.0.0, Dplyr version 0.7.8, Matrix version 1.2.15, Data.table version 1.11.8, Grid.Extra version 2.3, Ggplot2 version 3.1.0, MAST version 1.8.2, Reshape2 version 1.4.3, Limma version 3.38.3, Uwot version 0.1.3, Stats version 3.5.1.<br>All the statistical analysis except CITE-seq were performed using GraphPad Prism v8.3.0. Graphs and plots were also generated using GraphPad Prism v8.3.0 or ggplot2 package in R.<br>All the statistical analyses are two-sided. All correlations are rank-based spearman's correlations in which r represent Spearman's r and p represent two-tailed p values.

For manuscripts utilizing custom algorithms or software that are central to the research but not yet described in published literature, software must be made available to editors/reviewers. We strongly encourage code deposition in a community repository (e.g. GitHub). See the Nature Research guidelines for submitting code & software for further information.

Data

Policy information about availability of data

All manuscripts must include a data availability statement. This statement should provide the following information, where applicable:

- Accession codes, unique identifiers, or web links for publicly available datasets
- A list of figures that have associated raw data
- A description of any restrictions on data availability

The single-cell RNAseq data are available in the NCBI Gene Expression Omnibus (GSETBD). The accession number is GSE138156. Codes used to analyze data and generate figures are available on Github.

Field-specific reporting

Please select the one below that is the best fit for your research. If you are not sure, read the appropriate sections before making your selection.

☒ Life sciences ☐ Behavioural & social sciences ☐ Ecological, evolutionary & environmental sciences

For a reference copy of the document with all sections, see nature.com/documents/nr-reporting-summary-flat.pdf

Life sciences study design

All studies must disclose on these points even when the disclosure is negative.

Sample size	No statistical test was used to determine the number of samples. Sample sizes were determined as appropriate to evaluate detection of large vaccine effects. Cost was an important consideration.<br>The number of samples for the CITE-seq and re-challenge experiments were not considered prior to the start of the study. The sample sizes were chosen based on the protection from the first set of challenges and the numbers deemed appropriate to gain confidence/statistical significance in the results.
Data exclusions	At week 82, serum could not be collected from 2 animals in the SOSIP/3M-052 group. Therefore, 2 samples (RBi16 and RSf16) were not available for any analysis from that time point.<br>As a quality control, cells were filtered out from the CITE-seq analysis if they expressed fewer than 100 genes or had a mitochondrial gene content greater than 4 standard deviations above the median.
Replication	Replication of the study has not been verified in the traditional context. However, our results were highly consistent with and built up on the premises of recent studies in the field (Pauthner, et al. Immunity 2019 and Petitdemange, et al. JCI Insight 2019).
Randomization	Animals were randomly allocated based on age and Mamu-A*01 status.
Blinding	Investigators were not blinded in any experiment. We followed the immune responses closely after each immunization. We did not blind the investigators to be able to understand the immune responses and adjust the study protocols, as required.

Reporting for specific materials, systems and methods

We require information from authors about some types of materials, experimental systems and methods used in many studies. Here, indicate whether each material, system or method listed is relevant to your study. If you are not sure if a list item applies to your research, read the appropriate section before selecting a response.

Materials & experimental systems

n/a	Involved in the study	
☐	☒	Antibodies
☐	☒	Eukaryotic cell lines
☒	☐	Palaeontology
☐	☒	Animals and other organisms
☒	☐	Human research participants
☒	☐	Clinical data

Methods

n/a	Involved in the study	
☒	☐	ChIP-seq
☐	☒	Flow cytometry
☒	☐	MRI-based neuroimaging

Antibodies

Antibodies used	Antibody clone #, Conjugate, Supplier, Catalogue #, Lot #, Experiment, Dilution/concentration (as applicable, see methods for more details)<br>Ki67 B56, FITC, BD Biosciences, 556026, 7296502, T cell phenotyping, 10 in 100 µl<br>CD4 OKT4, Brilliant violet 605, BioLegend, 317438, B289706, ICS assay, 1.5 in 100 µl<br>CD4 OKT4, PerCP, BioLegend, 317432, B175630, T cell phenotyping, 3 in 100 µl<br>CD4 OKT4, Alexafluor647, BioLegend, 317422, B252657, IHC, 1:100 µl<br>CD28 28.2, PE Dazzle 594, BioLegend, 302942, B259245, T cell phenotyping, 2 in 100 µl<br>CD28 28.2, Purified NA/LE, BD Biosciences, 555725, 7075744, Stimulation, 1 µg/ml<br>CD49d 9F10, Purified NA/LE, BD Biosciences, 555501, 7075740, Stimulation, 1 µg/ml<br>CD127 eBioRDR5, PE-Cy7, eBioSciences, 25-1278-41, 4347271, T cell phenotyping, 1 in 100 µl<br>CCR7 G043H7, Brilliant violet 421, BioLegend, 353208, B249679, T cell phenotyping, 2 in 100 µl<br>CXCR3 G025H7, Brilliant violet 510, BioLegend, 353726, B235956, T cell phenotyping, 2.5 in 100 µl<br>CD95 DX2, Brilliant violet 605, BioLegend, 305628, B251257, T cell phenotyping, 2 in 100 µl<br>CD45RA MEM-56, Brilliant violet 650, Life Technologies, Q10069, 1465020, T cell phenotyping, 0.1 in 100 µl<br>CD8a RPA-T8, PerCP-Cy5.5, BioLegend, 301032, B271242, ICS assay, 3 in 100 µl<br>CD8a RPA-T8, Brilliant violet 711, BioLegend, 301044, B237121, T cell phenotyping, 2.5 in 100 µl<br>CD8a SK1, Alexafluor488, BioLegend, 344716, B279545, IHC, 1:100 µl

CD3 SP34-2, PE-CF594, BD Biosciences, 562406, 9240898, ICS assay, 0.5 in 100 µl
CD3 SP34-2, Alexafluor700, BD Biosciences, 557917, 8067751, T cell phenotyping, 2 in 100 µl
Granzyme-B GB12, PE, Life Technologies, MHGB04, 1990360, T cell phenotyping, 0.5 in 100 µl
IFN-γ 4S.B3, Alexafluor700, BioLegend, 502520, B243153, ICS assay, 1 in 100 µl
TNF-α Mab11, PE-Cy7, eBioSciences, 25-7349-82, E07679-1634, ICS assay, 0.3 in 100 µl
IL-2 MQ1-17H12, FITC, BioLegend, 500304, B231174, ICS assay, 2.5 in 100 µl
IL-4 8D4-8, PE, BioLegend, 500710, 5180219131, ICS assay, 5 in 100 µl
CD40L 24-31, Brilliant violet 711, 310838, B272545, ICS assay, 2.5 in 100 µl
CD69 FN50, PerCP-Cy5.5, BioLegend, 310926, B292697, ICS assay, 5 in 100 µl
MIP-1B D21-1351, BD Biosciences, 561120, ICS assay, 5 in 100 µl
CD45 D058 – 1283, FITC, BD Biosciences, 557803, 8138683, Livecell sorting, 10 in 100 µl
CD4 OKT-4, Totalseq-A0922, BioLegend, Custom conjugate, B284065, CITE-seq, 2 in 100 µl
CD8 RPA-T8, Totalseq-A0080, BioLegend, 301067, B268768, CITE-seq, 2 in 100 µl
CD20 2H7, Totalseq-A0100, BioLegend, 302359, B278747, CITE-seq, 2 in 100 µl
HLA-DR L243, Totalseq-A0159, BioLegend, 307659, B273589, CITE-seq, 2 in 100 µl
CD14 M5E2, Totalseq-A0081, BioLegend, 301855, B269442, CITE-seq, 2 in 100 µl
CD16 3G8, Totalseq-A0083, BioLegend, 302061, B271398, CITE-seq, 2 in 100 µl
CD11c S-HCL-3, Totalseq-A0053, BioLegend, 371519, B270802, CITE-seq, 2 in 100 µl
CD123 6H6, Totalseq-A0064, BioLegend, 306037, B265914, CITE-seq, 2 in 100 µl
CD1c L161, Totalseq-A0160, BioLegend, 331539, B271540, CITE-seq, 2 in 100 µl
CD69 FN50, Totalseq-A0146, BioLegend, 310947, B272035, CITE-seq, 2 in 100 µl
CD28 CD28.2, Totalseq-A0386, BioLegend, 302955, B273643, CITE-seq, 2 in 100 µl
CD95 DX2, Totalseq-A0156, BioLegend, 305649, B277719, CITE-seq, 2 in 100 µl
Monkey serum IgA, purified, Alpha Diagnostics, 20014-1, 70043, Total IgA ELISA, 0.5 µg/ml
Monkey serum IgG, purified, Alpha Diagnostics, 20012-1, 70023, Total IgG ELISA 0.5 µg/ml
Anti-human/primate CCL5 capture antibody clone 21418, purified, R&D Systems, MAB678, JA1118071, CCL5 ELISA, 2 µg/ml
Human/primate CCL5 detection antibody, polyclonal, R&D Systems, BAF278, VC1019011, CCL5 ELISA, 0.2 µg/ml

Validation

All the antibodies used in the flow cytometry experiments were tested and confirmed cross-reactive to Rhesus macaque PBMCs in our previous experiments. The antibodies used in ELISAs and other assays were known to be cross-reactive as tested by the producers. See methods for the source of the antibodies.

Eukaryotic cell lines

Policy information about cell lines

Cell line source(s)

HEK293T: ATCC, CRL-11268.
TZM-bl: NIH-AIDS reagent program Cat # 8129
CHO cells: ATCC CCL-61

Authentication

The cell lines were authenticated using short-tandem repeat determination through Cell Authentication services of ATCC. Reports can be obtained on request.

Mycoplasma contamination

The cell lines were periodically examined for mycoplasma contamination using ATCC's cell authentication service and were negative.

Commonly misidentified lines
(See ICLAC register)

No commonly misidentified cell line was used in the study.

Animals and other organisms

Policy information about studies involving animals; ARRIVE guidelines recommended for reporting animal research

Laboratory animals

All animals used in the study were female Rhesus Macaques (Macaca mulatta) of India origin, aged 3 - 15. A descriptive table containing age and MHC-I alleles are provided in supplementary table 1.

Wild animals

Study did not involve wild animals.

Field-collected samples

Study did not involve samples collected from the field.

Ethics oversight

Animals were maintained as per NIH guidelines and all procedures were approved by the Institutional Animal Care and Use Committee of Emory University. Reported in the first paragraph of the methods section.

Note that full information on the approval of the study protocol must also be provided in the manuscript.

Flow Cytometry

Plots

Confirm that:

☒ The axis labels state the marker and fluorochrome used (e.g. CD4-FITC).

☒ The axis scales are clearly visible. Include numbers along axes only for bottom left plot of group (a 'group' is an analysis of identical markers).

☒ All plots are contour plots with outliers or pseudocolor plots.

☒ A numerical value for number of cells or percentage (with statistics) is provided.

Methodology

Sample preparation	PBMCs were prepared from blood collected in BD cell preparation tubes using a standard protocol optimized in the lab. Briefly, the blood samples were centrifuged at 2,000 g for 20 min at room temperature. Plasma was removed and the cells were resuspended in PBS. The cells were transferred to a 15 or 50 ml centrifuge tube, washed once with PBS. The red blood cells were lysed using ACK lysis buffer. The cells were washed thrice and resuspended in complete media for use.<br>For staining, 1 - 2 million cells were washed once with PBS and stained with a viability dye followed by staining with a surface antibody cocktail in 100 ul for 30 min at room temperature. The cells were then washed, fixed and permeabilized with cytofix/cytoperm buffer for 10 minutes. The permeabilized cells were stained with ICS antibodies in perm/wash buffer for 30 min at room temperature. Cells were then washed twice with perm/wash buffer and once with staining buffer before acquisition.<br>The vaginal tissues harvested from animals undergoing necropsy were shipped overnight in cold RPMI supplemented with 10% FBS and antibiotics. The vaginal tissue slices collected after stimulation with DMSO or the Gag peptide pool were minced and digested in 1 mg/ml collagenase type IV. They were then dissociated using a gentleMACS dissociator (Milteyni Biotec) with the settings to dissociate mouse spleen. The dissociation protocol was run three times, and the samples were filtered through 100 µm strainers. The cells were washed twice with Hank's balanced salt solution (HBSS) with 2% FBS. The single cell suspensions were stained with fixable viability dye in PBS followed by staining with a cocktail of oligo-labelled antibodies (shown in Supplementary Table 2) and FITC anti-CD45 (clone D058 – 1283) and PE-CF594 anti-CD3 antibodies.
Instrument	BD LSRII for analysis. BD Aria III for live-dead sorting
Software	BD FACSDiva V8.0
Cell population abundance	The primary objective of the sorting was to obtain viable cells for single-cell RNA sequencing. We used this step to enrich for non-T cells. Purity was not of relevance as we mixed 3000 - 6000 viable cells for CITE-seq single cell seuqencing.
Gating strategy	Live CD8 and CD4 T cells were selected in three straightforward steps. 1) Singlets obtained using FSC-A vs. FSC-H. 2) Live T cells were selected as live CD3+. 3) CD4 and CD8 T cells were selected as CD3+ CD4+ or CD3+ CD8+.

☒ Tick this box to confirm that a figure exemplifying the gating strategy is provided in the Supplementary Information.
